# Suppression of Expression Between Adjacent Genes Within Heterologous Modules in Yeast

**DOI:** 10.1534/g3.113.007922

**Published:** 2013-11-26

**Authors:** Tae J. Lee, Rasesh Y. Parikh, Joshua S. Weitz, Harold D. Kim

**Affiliations:** * School of Biology, Georgia Institute of Technology, Atlanta, Georgia 30332-0430; † School of Physics, Georgia Institute of Technology, Atlanta, Georgia 30332-0430

**Keywords:** bidirectional promoter, coexpression, heterologous gene expression, nucleosome positioning

## Abstract

Recent studies have shown that proximal arrangement of multiple genes can have complex effects on gene expression. For example, in the case of heterologous gene expression modules, certain arrangements of the selection marker and the gene expression cassette may have unintended consequences that limit the predictability and interpretability of module behaviors. The relationship between arrangement and expression has not been systematically characterized within heterologous modules to date. In this study, we quantitatively measured gene expression patterns of the selection marker (*KlURA3* driven by the promoter, *pKlURA*) and the gene expression cassette (GFP driven by the galactose-inducible *GAL1* promoter, *pGAL1*) in all their possible relative arrangements in *Saccharomyces cerevisiae*. First, we observed that *pKlURA* activity depends strongly on the relative arrangement and the activity of *pGAL1*. Most notably, we observed transcriptional suppression in the case of divergent arrangements: *pKlURA* activity was reduced when *pGAL1* was inactive. Based on our nucleosome occupancy data, we attribute the observed transcriptional reduction to nucleosome repositioning. Second, we observed that *pGAL1* activity also depends on the relative arrangement of *pKlURA*. In particular, strains with divergent promoters showed significantly different *pGAL1* activation patterns from other strains, but only when their growth was compromised by lack of uracil. We reasoned that this difference in *pGAL1* activation patterns arises from arrangement-dependent *pKlURA* activity that can affect the overall cell physiology (*i.e.*, cell growth and survival in the uracil-depleted condition). Our results underscore the necessity to consider ramifications of promoter arrangement when using synthetic gene expression modules.

Stable expression of a heterologous gene module in prokaryotic or eukaryotic systems is widely used for various biological applications, ranging from basic gene expression studies to gene therapies (Buckholz and Gleeson 1991; Terpe 2006; Jana and Deb 2005; Desai *et al.* 2010; Romanos *et al.* 1992; Frommer and Ninnemann 1995). The typical design of a heterologous module combines two genetic components: a selection marker and a gene expression cassette that are adjacently integrated into the genome of the desired system. The selection marker, consisting of a gene that is constitutively driven by its own promoter, allows the cell to survive a condition or environment that is otherwise deleterious. The gene expression cassette, consisting of a promoter driving the expression of the heterologous gene, can be customized to a specific goal of study. For example, the gene expression cassette may contain a reporter gene that detects gene expression, a therapeutic gene that corrects a mutated gene (Cavazzana-Calvo *et al.* 2000), or a pharmaceutical gene that can be produced *en masse* (Buckholz and Gleeson 1991). Traditionally, the gene expression cassette and the selection marker, each with its own intended function, are designed to be closely positioned, and the efficiency of heterologous gene expression is thought to be primarily determined by the strength of the promoter within the gene expression cassette.

However, the traditional view of heterologous gene expression is becoming increasingly challenged by studies demonstrating that transcriptional activity from the same promoter can be differentially affected by adjacent promoters. This phenomenon, known as promoter interaction, may be important in determining gene expression levels, particularly because proximal arrangement of genes can lead to unexpected gene expression dynamics (Shearwin *et al.* 2005; Mazo *et al.* 2007). For example, expression of a gene can be inhibited by adjacent transcriptional activity through a mechanism known as transcriptional interference. In transcriptional interference, RNA polymerase (RNAP) initiated from one promoter can occlude proper initiation of RNAP on the adjacent promoter (Adhya and Gottesman 1982), or RNAPs transcribing adjacent genes toward each other can lead to their collision (Callen *et al.* 2004; Prescott and Proudfoot 2002; Martens *et al.* 2004). These modes of transcriptional interference have been systematically studied with mathematical models (Palmer *et al.* 2009; Sneppen *et al.* 2005) and with synthetic gene circuits (Buetti-Dinh *et al.* 2009).

Interestingly, transcriptional interference alone is not sufficient to explain gene expression patterns of adjacent genes in eukaryotic genomes. For example, two gene expression cassettes inserted into the same locus in mammalian cells were shown to be more correlated than when inserted at different genomic sites, suggesting spatial extension of gene activation along the genome (Raj *et al.* 2006). Furthermore, a large fraction of adjacent genes in nature are shown to be temporally correlated (Batada *et al.* 2007; Michalak 2008; Purmann *et al.* 2007). In particular, divergent (pointing away from each other) genes show greater correlation in their temporal dynamics (coexpression) and in noise than those in serial (pointing in the same direction) or convergent (pointing toward each other) genes (Trinklein *et al.* 2004; Wang *et al.* 2011), suggesting arrangement-dependent coexpression between adjacent genes.

Recent studies have proposed several mechanisms to account for coexpression between divergent genes. One mechanism is inherent bidirectional transcription from a single active promoter (Xu *et al.* 2009) that has been demonstrated in a large fraction of promoters in yeast. Consistent with this notion, coexpression in *Arabidopsis thaliana* is shown to be the strongest in gene pairs whose intergenic distance is less than 400 bp, indicating the presence of a single promoter in this region (Chen *et al.* 2010). Intriguingly, coexpression can also be strong in serial arrangements, which suggests that an additional mechanism may counteract transcriptional interference. An attractive mechanism is shared chromatin domains that switch between euchromatin and heterochromatin states, in which adjacent genes may be simultaneously expressed or repressed (Batada *et al.* 2007).

Transcriptional interference and coexpression mechanisms suggest that heterologous gene expression may be subject to complex gene regulation. For example, activity of the gene expression cassette may interfere with that of the selection marker and vice versa. Furthermore, because the selection marker is often essential for cell growth, its expression level is inevitably linked with the general cell physiology. Therefore, modulation of its activity by the activity of the adjacent gene may make it difficult to interpret and predict gene expression output.

In this work, we systematically studied promoter interaction within heterologous modules. To achieve this, we rearranged the selection marker and the gene expression cassette of a commonly used heterologous module in all possible configurations of relative arrangement and direction. With these modules, we first asked how the selection marker activity is affected by the relative arrangement and the activity of the gene expression cassette. We also asked how the activity of the gene expression cassette is affected by the relative arrangement of the selection marker. In addressing the first question, we observed that the selection marker can be significantly suppressed with the gene expression cassette, but only in the divergent arrangement. To elucidate the underlying mechanism, we obtained nucleosome occupancy data that suggest repositioning of nucleosomes as a potential explanation for transcriptional suppression. In addressing the second question, we also observed unique expression patterns in the gene expression cassette only in the divergent arrangement. Such unique expression patterns are likely attributable to the effect of the selection marker activity on the overall cell physiology.

## Materials and Methods

### Construction of gene circuits

All heterologous modules were derived from the plasmid pFA6a-*TRP1* (Longtine *et al.* 1998). The entire plasmid excluding the *TRP1* gene was PCR-amplified with primers GCGGGGATCCGTCGACCTGCAGCGTACGAA and GCGGCGAGCTCGAATTCATCGATG. The PCR-amplified fragment contains multiple enzyme-cutting sites including *Bam*HI and *Eco*RI. The *KlURA3* gene containing the 189-bp-long promoter region upstream of the *KlURA3* coding region was PCR-amplified with primers GCGGGGATCCGAATTCAATGAAAGAGAGAGAGAGAAGC and GCGGGAATTCGGATCCAGATCTGGATCTATATCACGTGATTTGC. This *KlURA3* amplicon also contains *Bam*HI at one end and *Eco*RI at the other end, and these cutting sites were used to fuse the amplicon from pFA6a-*TRP1* with *KlURA3* to generate pFA6a-*KlURA3*. Then, we PCR-amplified *pGAL1*-GFP-*yADHt* from pFA6a-*TRP1-pGAL1*-GFP (Longtine *et al.* 1998) with primers GCGGGAATTCGGATCCAGATCTGTAAAGAGCCCCATTATCTTAGCC and GCGGGGATCCGAATTCGGTGTGGTCAATAAGAGCGACCTC. Importantly, the *pGAL1* promoter, derived from the *GAL1-10* promoter, is deleted of the *GAL10* promoter elements (Johnston and Davis 1984) and contains the 542 bp *GAL1* promoter region upstream of its start codon. The termination signal for the *ADH1* gene (*yADHt*) is placed downstream of the GFP protein for transcription termination. The amplified fragment contains *Bam*HI and *Eco*RI at both ends and was inserted into pFA6a-*KlURA3*, at *Bam*HI site to generate divergent pFA6a-*KlURA3-pGAL1*-GFP or serial pFA6a-*KlURA3*-GFP-*pGAL1*, and at *Eco*RI site to generate convergent pFA6a-*pGAL1*-GFP-*KlURA3* or serial pFA6a-GFP-*pGAL1-KlURA3*. All PCRs were performed with high-fidelity DNA polymerase (#F-540L; New England Biolabs).

### Strain preparation

The heterologous modules were integrated into the yeast strain *W303*. A euchromatic region on chromosome XVI (Flagfeldt *et al.* 2009) was selected as the site of integration [site 20 in (Flagfeldt *et al.* 2009)] for minimal interference in gene expression from neighboring genomic regions. The heterologous modules were PCR-amplified with primers **GTAGTTTTAAAATTTCAAATCCGAACAACAGAGCATAGGGTTTCGCAAA***GTGGATCTGATATCATCGATG* and **GAGTTCTGTATTGTTCTTCTTAGTGCTTGTATATGCTCATCCCGACCTTCCATT***ACGCTGCAGGTCGACGGATC*. The italicized sequences are used for PCR-amplifying the heterologous modules and the bold sequences represent the genomic site of integration; they overlap with the genomic DNA for homologous recombination. As controls, **GTAGTTTTAAAATTTCAAATCCGAACAACAGAGCATAGGGTTTCGCAAA***AATGAAAGAGAGAGAGAGAAGC* and **GAGTTCTGTATTGTTCTTCTTAGTGCTTGTATATGCTCATCCCGACCTTCCATT***GGATCTATATCACGTGATTTGC* were used to PCR-amplify the *KlURA3* gene only. To create strains with the modules integrated in the reverse direction, the italicized sequences were switched between the two primers, whereas the bold sequences remained the same. The standard LiAc/SS carrier DNA/PEG method was used for high-efficiency yeast transformation (Gietz and Schiestl 2007), and transformed colonies were selected on synthetic defined (SD) complete plates with 2% glucose minus uracil (#100217-544; Teknova). The genomic integration of heterologous modules was verified by PCR amplification. The promoter regions of our divergent promoter strains were sequenced and no mutation was detected over the *pKlURA* and *pGAL1* promoter regions (Supporting Information, Table S6).

### Culture conditions and growth assay

Before induction, cells were grown in liquid synthetic raffinose (2%) medium with additional 0.05% glucose (minimal amount to ensure even growth between different cell strains) (Acar *et al.* 2010) overnight for approximately 16 hr. The next day, these cells were inoculated into fresh media for exponential growth. Cells in exponential growth phase were then re-inoculated into synthetic raffinose (2%) medium with additional 0.05% glucose lacking uracil in the absence or presence of 2% galactose. After 24 hr of growth, cells were then transferred onto a 96-well plate for growth assays (Synergy H1; Biotek). The optical density (OD_600_) was measured every 30 min for 6 hr, with 4 min of linear shaking immediately before measurements.

### Quantification of KlURA3 transcript

The total mRNA was obtained from divergent and control strains after 24 hr of galactose induction or no induction. Cells were grown in 5 ml media [synthetic raffinose (2%) medium with 0.05% glucose lacking uracil] to logarithmic growth phase. At OD_600_ ∼0.6, these cells were harvested and homogenized with TRIzol (#15596-026; Ambion). Cells were vortexed with acid beads (#12621-152; VWR) for 1 hr at 4° followed by mRNA precipitation. The precipitated mRNA was treated with DNase I (#AM2222; Ambion) and was converted to cDNA with a kit from Invitrogen (#18080-400, SuperScript III First-Strand Synthesis SuperMix). The amount of cDNA was measured by standard quantitative real-time PCR (qPCR) with a PCR master mix (#4309155; Applied Biosystem). For *KlURA3* quantification, primers CCGTGGGCGTTGGTGATATC and GAGGGTACTGTCGTTCCATTG were used. As control, the *ACT1* gene was targeted with primers TGTCACCAACTGGGACGATA and AACCAGCGTAAATTGGAACG (Kessler *et al.* 2003).

### Nucleosome mapping

The standard nucleosome scanning assay was performed as described (Infante *et al.* 2012). Briefly, divergent and control yeast cultures (100–200 ml) were grown for 24 hr in Gal^−^ and Gal^+^ conditions. At OD_600_∼0.6, they were fixed with formaldehyde, treated with zymolyase for conversion of cells to osmotically fragile spheroplasts, and digested with varying concentrations of micrococcal nuclease (catalog #M0247S; New England BioLabs). The resulting mononucleosomal DNA was isolated and purified, and then was used to map nucleosome positions in chromatin by qPCR (#4309155; Applied Biosystem). The primer sets used in this study are listed in Table S7. For each primer set, a standard curve was obtained by serial dilution of the genomic DNA for both control and divergent-promoter strains. Using the standard curves, the relative concentration of mononucleosomal DNA (normalized to the reference genomic position) was calculated.

### Statistical analysis

Paired *t* tests were performed with the Matlab statistics toolbox to compare the growth patterns or the nucleosome occupancy between two different strains. Each paired *t* test was based on at least three independent experiments. The growth rates were obtained by estimating the slope of a line fit to a semi-log plot of the growth curves (Table S1). Then, these growth rates between different strains were compared for statistical significance. For nucleosome occupancy, the control strain was compared with the uninduced or induced case of the divergent strain for statistical significance. The *t* tests with p-value < 0.05 are considered to show a statistically significant difference between the compared strains.

## Results

### Construction of all pairwise arrangements of elements in a heterologous module

**Figure 1:**
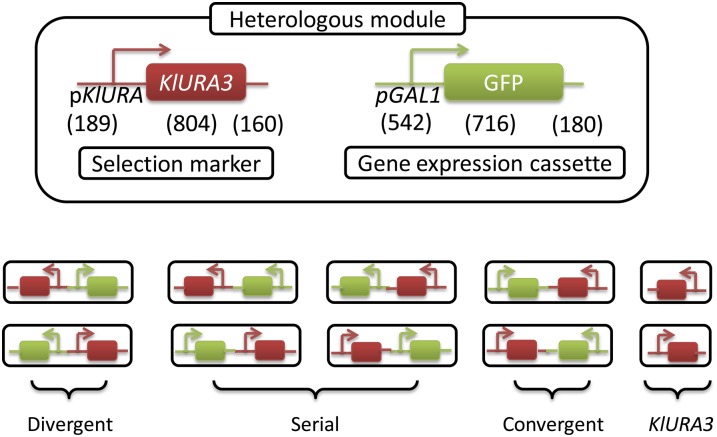
Construction of all pairwise arrangements of heterologous gene expression modules. Our heterologous modules consist of two functionally unrelated genes: the *KlURA3* gene from *K. lactis* with its own promoter (*pKlURA*) driving the downstream protein coding region (represented by the red box) with the native flanking region (represented by the red line) and the *GAL1* promoter (*pGAL1*) driving the expression of GFP (represented by the green box) with the *ADH1* terminator signal (represented by the green line). The length of each element (in bp) is indicated. These genes are adjacently placed in various arrangements: bidirectional (transcribing away from each other), serial (transcribing in the same direction), and convergent (transcribing in the opposite direction). The control strains consist of only the *KlURA3* gene. These modules were integrated in both 5′ to 3′ (top row) and 3′ to 5′ directions (bottom row) into the budding yeast genome.

We first constructed heterologous modules of all possible arrangements between a selection marker and a gene expression cassette as shown in Figure 1. The selection marker consists of the *URA3* promoter (*pKlURA*) from *Kluyveromyces lactis* and its downstream protein-coding region *KlURA3*. The *KlURA3* gene encodes the enzyme orotidine 5′-phosphate decarboxylase responsible for the synthesis of uracil, which is one of the nucleobases for mRNA. Thus, active *pKlURA* is essential for cell growth in media lacking uracil (URA^−^ condition). The gene expression cassette consists of the galactose-inducible *GAL1* promoter (*pGAL1*) driving the expression of GFP (*pGAL1*-GFP). This *pGAL1* promoter is derived from the divergent *pGAL1-10* promoter by removing a significant portion of the *cis*-regulatory elements of *pGAL10* (126 bp upstream of the coding region of the *GAL10* gene). Such removal has been shown to disable bidirectional transcription and to allow gene expression only in the 5′ to 3′ direction in the derived *GAL1* promoter (Johnston and Davis 1984). The *pKlURA* and *pGAL1* promoters are widely used in yeast experiments and do not share common *cis*-regulatory elements or *trans*-factors. Whereas *pKlURA* is expected to be always active in the Ura**^−^** condition, *pGAL1* is known to exhibit a wide dynamic range, up to 1000-fold change, in gene expression level (Johnston and Davis 1984) depending on galactose induction. Thus, the effect of *pGAL1* activity on *pKlURA* can be analyzed for a wide range of galactose concentrations. These heterologous modules were inserted into the yeast genome. The resulting yeast strains are named after the heterologous modules they carry (*i.e.*, a divergent strain is defined as a yeast strain that carries the divergent module). As controls, we constructed strains carrying only *KlURA3*. All our modules were integrated at a transcriptionally “neutral” site in the yeast genome (Flagfeldt *et al.* 2009), where the gene expression of the inserted construct was the highest compared to other genomic sites investigated. We expected that, at this site, neighboring genomic elements would not significantly affect the expression of heterologous modules. To experimentally confirm this, we genomically integrated our heterologous modules in both 5′ to 3′ and 3′ to 5′ directions in *Saccharomyces cerevisiae*.

### Divergent arrangement leads to significantly smaller colony size

We first grew our strains on SD agar plates lacking uracil with glucose as the carbon source. In this condition, the growth of cells depends on their ability to synthesize uracil (via *pKlURA* activity). Interestingly, we observed a striking difference in colony size between our strains (Figure S1A). The colony size of divergent promoter strains was significantly smaller than that of serial or convergent promoter strains. This was also shown in our serial dilution spotting assays in Figure S1B. We note that these strains are genetically identical except for the relative arrangement of *KlURA3* and *pGAL1*-GFP. Therefore, we reasoned that the difference in colony size was because of arrangement-dependent *pKlURA* activity. In support of this reasoning, when our strains were grown on SD agar plates supplemented with uracil (Ura**^+^**), the difference in colony size was nearly eliminated (Figure S1A). These results suggest that the amount of uracil synthesized by cells in the Ura^−^ condition (or *pKlURA* activity) is the limiting factor for cell growth and, thus, growth rates can be used as a proxy for pKlURA activity.

For more quantitative comparison of *pKlURA* activity between different strains, we measured their growth rates in liquid synthetic raffinose media lacking uracil. Using the growth rate as a proxy for *pKlURA* activity, we first tested whether the genomic site of integration was neutral to neighboring genomic regions. Validation of “neutrality” was important to ensure that the neighboring genomic elements do not significantly affect the transcriptional activity of *pGAL1* and *pKlURA*. Our growth assays showed that the two control strains, which differ only in the directionality of integration, exhibited similar growth curves. We quantitatively compared these strains by performing paired *t* tests of their growth rates (extracted from their growth curves) (Table S1). Our results showed no statistically significant difference between the two control strains (Figure S2 and Table S2). These results suggest that gene expression at this genomic site is not considerably biased by either the upstream or the downstream genomic elements, and that the neighboring genomic regions do not significantly influence the behavior of *pGAL1* and *pKlURA*.

### Relative position of pGAL1 determines pKlURA activity

**Figure 2:**
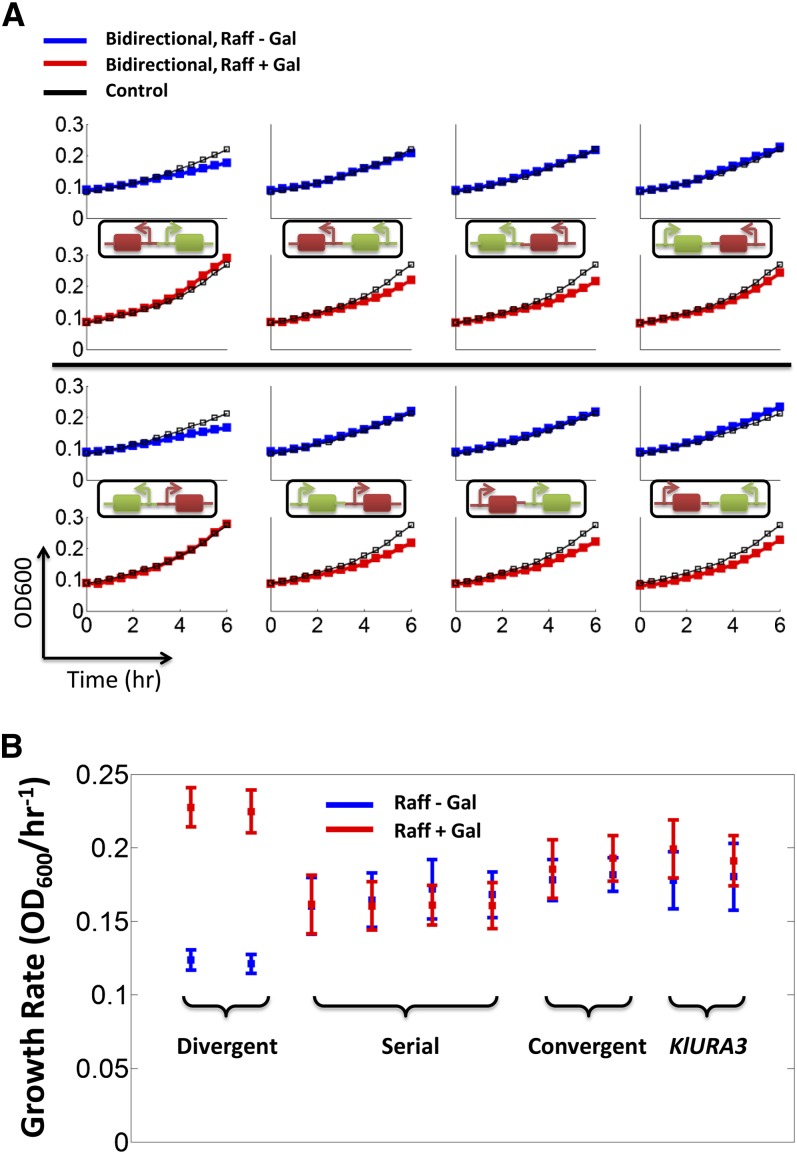
Modulation of *pKlURA* activity by galactose induction. After 24 hr of growth without uracil in the Gal^−^ or Gal^+^ condition, a small portion of cells were moved to fresh media on a 96-well plate and their growth at steady state was measured for 6 hr at 30-min intervals with a plate reader. (A) Growth curves, as measured by OD_600_, of the designated strains. Control strains grown in either condition are plotted as black lines with square boxes for comparison. The growth curves in the GAL^−^ or GAL^+^ conditions are denoted by blue or red lines with square boxes, respectively. A representative set of experiments is shown here. Additional set of growth experiments can be found in Figure S3. The top and bottom panels differ in the direction of genome integration. The growth curves reflect “raw” OD_600_ measurements on a plate reader and are not corrected by the baseline OD_600_. (B) Growth rates extracted from the growth curves. The error bars represent at least three independently measured growth rates. The growth rates in the Gal^−^ or Gal^+^ condition are denoted by blue or red squares, respectively.

Next, we compared basal growth patterns of all our strains in liquid synthetic raffinose media lacking uracil without galactose induction (Gal^−^). If *pGAL1*-GFP and *KlURA3* were transcriptionally independent, we would not expect differences in growth patterns between strains. Consistent with this notion, our convergent and serial promoter strains showed similar growth rates as control strains (blue in Figure 2, A and B and Figure S3). Interestingly, however, we observed a significant decrease in growth rate in divergent promoter strains compared to other strains (see Table S3 for statistical analyses). We note that the observed decrease in growth rate (or repressed *pKlURA* activity) is unlikely to be explained by transcriptional interference via RNAP, given that *pGAL1* is known to be tightly repressed when not induced (Johnston and Davis 1984). This is also evidenced by our measurement of basal GFP level without galactose induction (Figure S4). When our strains were supplemented with uracil (Ura^+^), they showed similar growth patterns (Figure S5), consistent with our growth assay on agar plates in Figure S1. We concluded that the relative arrangement of *pKlURA* and *pGAL1* can account for the variable colony size and growth rate, given that the relative arrangement is the only difference between our strains.

### Activity of pGAL1 determines pKlURA activity

We also measured changes in growth rate on *pGAL1* induction by adding galactose (Gal^+^) (red in Figure 2, A and B and Figure S3). In this condition, we observed three distinct growth patterns. First, we observed a minor increase in growth rate on galactose induction (in convergent and control strains). This was expected for all cell strains because galactose, being a carbon source, can improve overall cell growth. However, our serial promoter strains did not show a significant change in growth rate between Gal^−^ and Gal^+^ conditions, reminiscent of transcriptional interference in the serial arrangement of promoters (Shearwin *et al.* 2005; Sneppen *et al.* 2005). Finally, we observed an increase in growth rate in divergent promoter strains, and this increase was significantly larger than the minor increase attributable to galactose addition (Table S3). We discuss potential explanations in the *Discussion* section. These results together suggest that *pGAL1* activity can control *pKlURA* activity when divergently arranged.

Thus far, we have assumed that growth rate can be used as a proxy for *pKlURA* activity, and that *pKlURA* activity can be extrapolated from the growth rate. To validate this assumption, we quantified the *KlURA3* transcript level in the same experimental conditions as growth assays, in the absence of galactose and in the presence of 2% galactose (Table S4). Consistent with our growth assays, our results showed that *pKlURA* activity in divergent promoter strains is nearly halved in comparison to the control strains without galactose and doubled with galactose induction. Our growth assay and *KlURA3* transcript quantification results demonstrate that *pKlURA* transcription level is positively correlated to the growth rate, although the relationship may be nonlinear.

### Modulation of pKlURA activity by pGAL1 may be mediated by repositioning of nucleosomes

**Figure 3:**
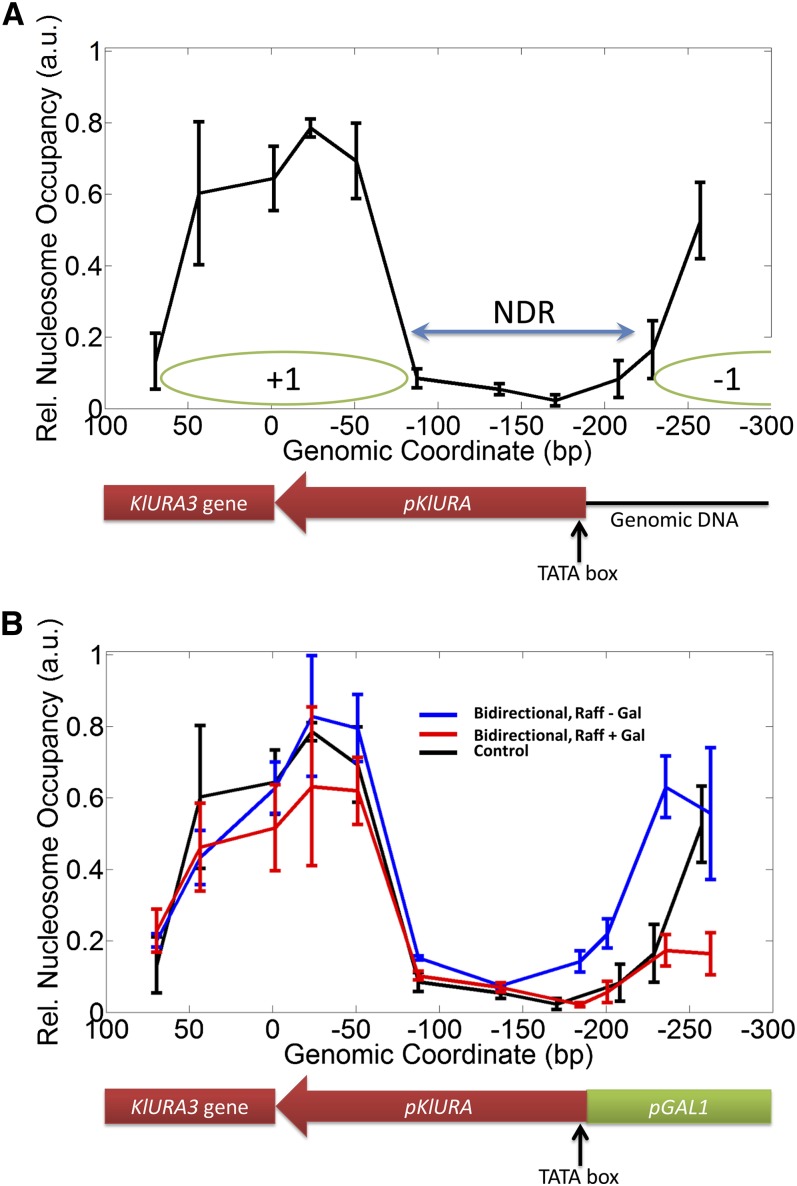
Repositioning of nucleosomes over *pKlURA* in the bidirectional strain. The standard nucleosome scanning assay (Infante *et al.* 2012) with micrococcal nuclease was used to isolate and purify mononucleosomal DNA. This mononucleosomal DNA was used to measure the relative nucleosome occupancy at the designated genomic positions. (A) The nucleosome occupancy in the control strain over *pKlURA* in Gal^−^ condition is plotted. The nucleosome-depleted region (NDR) is indicated by the blue arrow. The green ovals denote strongly positioned nucleosomes (+1 and −1) around the NDR. The *KlURA3* promoter region and the downstream protein-coding region are indicated by the red arrow and the red box, respectively. (B) The nucleosome occupancy in the bidirectional strain is mapped in Gal^−^ (red) and Gal^+^ (blue) conditions. The nucleosome occupancy in the control strain is overlaid for comparison (black). In both plots, the x-axis represents the genomic coordinate relative to the start codon (ATG) of the *KlURA3* gene. The *pGAL1* region is denoted by the green arrow. Error bars represent three independent measurements of relative nucleosome occupancy.

To explain transcriptional suppression, we measured nucleosome occupancy over *pKlURA* in Gal^−^ and Gal^+^ conditions. First, we mapped nucleosome occupancy over *pKlURA* in the control strain (Figure 3A). In the control strain, we did not expect galactose to have an effect on the nucleosome occupancy over *pKlURA*. As expected, our results did not show a significant difference between Gal^−^ and Gal^+^ conditions (Figure S6). The nucleosome occupancy pattern resembles that of its orthologous URA3 promoter (Jansen *et al.* 2012): strongly positioned −1 and +1 nucleosomes of ∼147 bp surrounding the nucleosome-depleted region (NDR) of ∼100 bp. The −1 nucleosome is positioned over the genomic region immediately next to the NDR region of *pKlURA*, and the +1 nucleosome is centered around the start codon of the *KlURA3* gene. The putative TATA signal (Mizukami and Hishinuma 1988) resides immediately next to *pGAL1* (∼185 bp upstream of the start codon of *KlURA3*).

Then, we also mapped nucleosome occupancy over *pKlURA* in the divergent strain and performed statistical analysis to compare with the control strain in Gal^−^ and Gal^+^ conditions (Figure 3B and Table S5). Without galactose, our results showed that the divergent placement of *pGAL1* generally increases nucleosome occupancy over *pKlURA* (blue line in Figure 3B) relative to the control strain. In particular, we observed a significant increase in the nucleosome occupancy over the putative TATA signal. Such increase in nucleosome occupancy is generally associated with transcriptional repression (Shivaswamy *et al.* 2008) and may account for the repressed *pKlURA* activity in the Gal^−^ condition. With galactose induction, the nucleosome occupancy over *pGAL1* is substantially lowered (red line in Figure 3B), resulting in exposure of the TATA signal on *pKlURA*. Such exposure would “restore” *pKlURA* activity to the level observed in the control strain but is insufficient to account for the two-fold increase in the *KlURA3* transcript level. Possible explanations are provided in the *Discussion* section.

### pGAL1 activation patterns in divergent arrangements are significantly different from those in other cases

**Figure 4:**
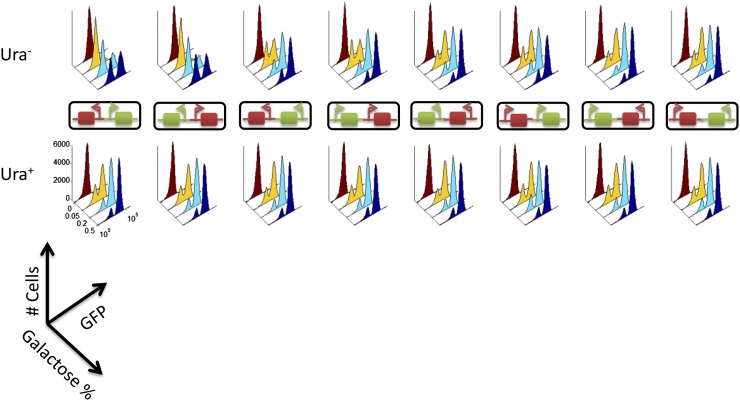
Unique GFP patterns in bidirectional strains. After 24 hr of growth in various galactose concentrations (0%, 0.05%, 0.2%, and 0.5%), cells were washed, fixed, and measured for their GFP intensity with flow cytometry. GFP histograms of the designated strains grown in the absence (top) or presence (bottom) of uracil are shown. Each histogram represents the GFP measurement of 50,000 cells.

Finally, we measured *pGAL1* activity in varying concentrations of galactose in the Ura^−^ condition (top histograms in Figure 4). The galactose signaling pathway is known to switch on in an all-or-none manner (Acar *et al.* 2005). Consistent with this, *pGAL1* activity remained low, with all cells expressing only the basal level of GFP without galactose induction. With increasing galactose concentration, *pGAL1* activity in all our strains switched from the low mode to the high mode of GFP (ON cells) in a bimodal manner. We separated the low and high modes of GFP using a Gaussian Mixture Model and calculated the fraction of ON cells (Figure S7A) and their mean GFP level (Figure S7B). Interestingly, we observed that *pGAL1* activation patterns in divergent promoter strains are significantly different from other strains. For example, only ∼25% of divergent cells showed *pGAL1* activation at 0.2% galactose, whereas ∼75% of serial and convergent cells showed *pGAL1* activation at the same galactose concentration. Furthermore, the mean GFP level was nearly twice as high in divergent promoter strains as in other strains.

We suspected that the observed difference in *pGAL1* activation patterns may be attributable to different growth rates arising from arrangement-dependent *pKlURA* activity. To test this, we measured *pGAL1* activity of all our strains in the Ura^+^ condition (bottom panels in Figure 4). In this condition, we showed that the growth rate is nearly identical between strains (Figure S5). With uracil supplemented, the difference in the fraction of ON cells and their mean GFP levels was nearly eliminated (bottom histograms in Figure 4 and Figure S7, A and B). Interestingly, similar *pGAL1* activation patterns were also observed without uracil supplemented in the growth media, as long as cells were grown at a similar rate with sufficiently strong galactose induction. Such growth rate–dependent *pGAL1* activation patterns illustrate how the relative arrangement of *pGAL1* and *pKlURA* can fine-tune the gene expression dynamics of both promoters.

## Discussion

We experimentally demonstrated that the relative arrangement of *pKlURA* and *pGAL1* has a profound impact on gene expression dynamics. Most notably, we observed that the activity of *pKlURA* can be altered when arranged with *pGAL1* in the divergent configuration. Compared to its normal level without *pGAL1*, the *pKlURA* activity is reduced when *pGAL1* is not induced. This reduction may be attributable to repositioning of nucleosomes in the *KlURA3* promoter region as a result of *pGAL1* arrangement. This idea is supported by previous studies performed by other groups. In one study, it was shown that *pGAL1* contains nucleosome-phasing elements (Floer *et al.* 2010) that may reposition neighboring nucleosomes. In another study, when *pURA3* was placed in various genomic contexts, the “strongly” positioned nucleosomes were either repositioned or even removed (Jansen *et al.* 2012). These results suggest that nucleosome positioning strongly depends on the nearby genomic elements. Consistent with these studies, our nucleosome mapping also showed that a nucleosome becomes repositioned closer toward the putative TATA box of the *KlURA3* promoter without galactose induction, likely accounting for reduced *pKlURA* activity.

In the same divergent configuration, we also observed that the *pKlURA* activity is higher than normal when *pGAL1* is induced. This increased *pKlURA* activity with galactose induction can be accounted for by the recruitment of *trans*-factors involved in *pGAL1* activation in the vicinity of *pKlURA*. This idea is supported by the fact that *pGAL1* is taken from the divergent *pGAL1-10* promoter that is known to be intrinsically bidirectional in its transcriptional activity. These *trans*-factors can be general transcription factors, chromatin remodeling complexes, or histone-modifying complexes. Widespread bidirectional transcription among yeast promoters is also a possibility (Xu *et al.* 2009; Churchman and Weissman 2011; Trinklein *et al.* 2004; Chen *et al.* 2010). Further experimental investigation is warranted to identify the *trans*-factors that spread from *pGAL1* to the *pKlURA*.

The activity of *pGAL1* was also different in divergent promoter strains compared to serial and convergent promoter strains. However, the change in *pGAL1* activity only occurred in uracil starvation and was more complicated than would be expected from simple crosstalk between promoters; whereas the fraction of ON cells decreased, the mean expression level of the ON cells increased (Figure S7). Given nearly identical GFP activation patterns when cells grow at similar rates (either by uracil supplement or by strong galactose induction), we suggest that the unique GFP activation patterns are likely attributable to the global effects of growth rate on gene expression (Klumpp *et al.* 2009). These results suggest that the relative arrangement between the selection marker and the gene expression cassette is an important parameter in the design of heterologous gene expression modules.

Regardless of the exact mechanism, we showed that two functionally unrelated promoters can be transcriptionally coupled in the divergent arrangement. It is worth noting that naturally occurring divergent promoters are distinguished from our divergent promoters in that they may belong to similar categories of gene function (Wakano *et al.* 2012) and often share common *cis*-regulatory or *trans*-regulatory elements (Trinklein *et al.* 2004). The mode of interaction in divergent promoters can be quite diverse. Coexpression can be explained by bidirectional transcription attributable to various factors, including common *cis*-regulatory elements (in *MAL6T-MAL6S*) (Bell *et al.* 1995) or chromatin organization (in *UGA3-GLT1*) (Ishida *et al.* 2006). In contrast, some studies reported no dependence on gene orientation (Bae *et al.* 2008) and others reported anticorrelated gene expression between divergent genes (Lin *et al.* 2007). Multiple factors may account for the differences in how divergent promoters behave, including the structure and properties of promoters (*i.e.*, noisy *vs.* robust or strong *vs.* weak) and how they are expressed in the cell (*i.e.*, genomically *vs.* from plasmids). In addition, the genomic site of integration may be an important parameter that governs whether the inserted promoters can affect or be affected by the neighboring genes. Therefore, generalization of transcriptional coupling would require rigorous and systematic characterization of promoters and experimental conditions.

In summary, we propose that any inducible promoter with elements that can affect its neighboring nucleosome positioning can be divergently fused to modulate its adjacent promoter activity. In fact, transcriptional coupling with an inducible promoter may be an alternative design principle for efficient control of gene expression with unique benefits. By externally controlling the chromatin state of *pGAL1*, we observed “overexpression” and “knockdown” of the target gene (*KlURA3* in our study) (Figure 2), which are approaches commonly used to study gene functions. The unique benefits of this method are that the expression of the target gene can be driven by its own native promoter, and its activity can be externally modulated around its native activity level. In other words, a constitutive promoter can be engineered to behave as an inducible promoter when divergently fused with an inducible promoter. Moreover, two inducible promoters that are divergently positioned may generate nontrivial gene expression patterns. Consistent with this notion, recent theoretical and bioinformatics studies suggested that divergent arrangement of two genes in proximity may be a noise-reducing mechanism (Wang *et al.* 2011; Woo and Li 2011). Mathematical models will be necessary to improve design and characterization of such promoter interactions.

## Supplementary Material

Supporting Information

## References

[bib1] Acar M. Becskei A. Van Oudenaarden A., 2005 Enhancement of cellular memory by reducing stochastic transitions. Nature 435: 228–232. 15889097 10.1038/nature03524

[bib2] Acar M. Pando B. F. Arnold F. H. Elowitz M. B. Van Oudenaarden A., 2010 A general mechanism for network-dosage compensation in gene circuits. Science 329: 1656–1660. 20929850 10.1126/science.1190544 PMC3138731

[bib3] Adhya S. Gottesman M., 1982 Promoter occlusion: transcription through a promoter may inhibit its activity. Cell 29: 939–944. 6217898 10.1016/0092-8674(82)90456-1

[bib4] Bae J. Y. Laplaza J. Jeffries T. W., 2008 Effects of gene orientation and use of multiple promoters on the expression of XYL1 and XYL2 in Saccharomyces cerevisiae. Appl. Biochem. Biotechnol. 145: 69–78. 18425613 10.1007/s12010-007-8076-0

[bib5] Batada N. N. Urrutia A. O. Hurst L. D., 2007 Chromatin remodelling is a major source of coexpression of linked genes in yeast. Trends Genet. 23: 480–484. 17822800 10.1016/j.tig.2007.08.003

[bib6] Bell P. J. Bissinger P. H. Evans R. J. Dawes I. W., 1995 A two-reporter gene system for the analysis of bi-directional transcription from the divergent MAL6T–MAL6S promoter in Saccharomyces cerevisiae. Curr. Genet. 28: 441–446. 8575017 10.1007/BF00310813

[bib7] Buckholz R. G. Gleeson M. A., 1991 Yeast systems for the commercial production of heterologous proteins. Biotechnology (N. Y.) 9: 1067–1072. 1367623 10.1038/nbt1191-1067

[bib8] Buetti-Dinh A. Ungricht R. Kelemen J. Z. Shetty C. Ratna P., 2009 Control and signal processing by transcriptional interference. Mol. Syst. Biol. 5: 300. 19690569 10.1038/msb.2009.61 PMC2736658

[bib9] Callen B. P. Shearwin K. E. Egan J. B., 2004 Transcriptional interference between convergent promoters caused by elongation over the promoter. Mol. Cell 14: 647–656. 15175159 10.1016/j.molcel.2004.05.010

[bib10] Cavazzana-Calvo M. Hacein-Bey S. De Saint Basile G. Gross F. Yvon E., 2000 Gene therapy of human severe combined immunodeficiency (SCID)-X1 disease. Science 288: 669–672. 10784449 10.1126/science.288.5466.669

[bib11] Chen W. H. De Meaux J. Lercher M. J., 2010 Co-expression of neighbouring genes in Arabidopsis: separating chromatin effects from direct interactions. BMC Genomics 11: 178. 20233415 10.1186/1471-2164-11-178 PMC2851598

[bib12] Churchman L. S. Weissman J. S., 2011 Nascent transcript sequencing visualizes transcription at nucleotide resolution. Nature 469: 368–373. 21248844 10.1038/nature09652 PMC3880149

[bib13] Desai P. N. Shrivastava N. Padh H., 2010 Production of heterologous proteins in plants: strategies for optimal expression. Biotechnol. Adv. 28: 427–435. 20152894 10.1016/j.biotechadv.2010.01.005

[bib14] Flagfeldt D. B. Siewers V. Huang L. Nielsen J., 2009 Characterization of chromosomal integration sites for heterologous gene expression in Saccharomyces cerevisiae. Yeast 26: 545–551. 19681174 10.1002/yea.1705

[bib15] Floer M. Wang X. Prabhu V. Berrozpe G. Narayan S., 2010 A RSC/nucleosome complex determines chromatin architecture and facilitates activator binding. Cell 141: 407–418. 20434983 10.1016/j.cell.2010.03.048 PMC3032599

[bib16] Frommer W. B. Ninnemann O., 1995 Heterologous expression of genes in bacterial, fungal, animal, and Plant-cells. Annu. Rev. Plant Physiol. Plant Mol. Biol. 46: 419–444.

[bib17] Gietz R. D. Schiestl R. H., 2007 High-efficiency yeast transformation using the LiAc/SS carrier DNA/PEG method. Nat. Protoc. 2: 31–34. 17401334 10.1038/nprot.2007.13

[bib18] Infante J. J. Law G. L. Young E. T., 2012 Analysis of nucleosome positioning using a nucleosome-scanning assay. Methods Mol. Biol. 833: 63–87. 22183588 10.1007/978-1-61779-477-3_5

[bib19] Ishida C. Aranda C. Valenzuela L. Riego L. Deluna A., 2006 The UGA3–GLT1 intergenic region constitutes a promoter whose bidirectional nature is determined by chromatin organization in Saccharomyces cerevisiae. Mol. Microbiol. 59: 1790–1806. 16553884 10.1111/j.1365-2958.2006.05055.x

[bib20] Jana S. Deb J. K., 2005 Strategies for efficient production of heterologous proteins in Escherichia coli. Appl. Microbiol. Biotechnol. 67: 289–298. 15635462 10.1007/s00253-004-1814-0

[bib21] Jansen A. Van Der Zande E. Meert W. Fink G. R. Verstrepen K. J., 2012 Distal chromatin structure influences local nucleosome positions and gene expression. Nucleic Acids Res. 40: 3870–3885. 22241769 10.1093/nar/gkr1311 PMC3351160

[bib22] Johnston M. Davis R. W., 1984 Sequences that regulate the divergent GAL1–GAL10 promoter in Saccharomyces cerevisiae. Mol. Cell. Biol. 4: 1440–1448. 6092912 10.1128/mcb.4.8.1440-1448.1984 PMC368932

[bib23] Kessler M. M. Zeng Q. Hogan S. Cook R. Morales A. J., 2003 Systematic discovery of new genes in the Saccharomyces cerevisiae genome. Genome Res. 13: 264–271. 12566404 10.1101/gr.232903 PMC420365

[bib24] Klumpp S. Zhang Z. Hwa T., 2009 Growth rate-dependent global effects on gene expression in bacteria. Cell 139: 1366–1375. 20064380 10.1016/j.cell.2009.12.001 PMC2818994

[bib25] Lin J. M. Collins P. J. Trinklein N. D. Fu Y. Xi H., 2007 Transcription factor binding and modified histones in human bidirectional promoters. Genome Res. 17: 818–827. 17568000 10.1101/gr.5623407 PMC1891341

[bib26] Longtine M. S. McKenzie A. 3rd DeMarini D. J. Shah N. G. Wach A., 1998 Additional modules for versatile and economical PCR-based gene deletion and modification in Saccharomyces cerevisiae. Yeast 14: 953–961. 9717241 10.1002/(SICI)1097-0061(199807)14:10<953::AID-YEA293>3.0.CO;2-U

[bib27] Martens J. A. Laprade L. Winston F., 2004 Intergenic transcription is required to repress the Saccharomyces cerevisiae SER3 gene. Nature 429: 571–574. 15175754 10.1038/nature02538

[bib28] Mazo A. Hodgson J. W. Petruk S. Sedkov Y. Brock H. W., 2007 Transcriptional interference: an unexpected layer of complexity in gene regulation. J. Cell Sci. 120: 2755–2761. 17690303 10.1242/jcs.007633

[bib29] Michalak P., 2008 Coexpression, coregulation, and cofunctionality of neighboring genes in eukaryotic genomes. Genomics 91: 243–248. 18082363 10.1016/j.ygeno.2007.11.002

[bib30] Mizukami M. Hishinuma F., 1988 Isolation and Nucleotide-Sequence Analysis of the Ura3 (Orotidine 5′-Phosphate Decarboxylase) Gene of Kluyveromyces-Lactis. Agric. Biol. Chem. 52: 3067–3071.

[bib31] Palmer A. C. Ahlgren-Berg A. Egan J. B. Dodd I. B. Shearwin K. E., 2009 Potent transcriptional interference by pausing of RNA polymerases over a downstream promoter. Mol. Cell 34: 545–555. 19524535 10.1016/j.molcel.2009.04.018 PMC2697128

[bib32] Prescott E. M. Proudfoot N. J., 2002 Transcriptional collision between convergent genes in budding yeast. Proc. Natl. Acad. Sci. USA 99: 8796–8801. 12077310 10.1073/pnas.132270899 PMC124378

[bib33] Purmann A. Toedling J. Schueler M. Carninci P. Lehrach H., 2007 Genomic organization of transcriptomes in mammals: Coregulation and cofunctionality. Genomics 89: 580–587. 17369017 10.1016/j.ygeno.2007.01.010

[bib34] Raj A. Peskin C. S. Tranchina D. Vargas D. Y. Tyagi S., 2006 Stochastic mRNA synthesis in mammalian cells. PLoS Biol. 4: e309. 17048983 10.1371/journal.pbio.0040309 PMC1563489

[bib35] Romanos M. A. Scorer C. A. Clare J. J., 1992 Foreign gene expression in yeast: a review. Yeast 8: 423–488. 1502852 10.1002/yea.320080602

[bib36] Shearwin K. E. Callen B. P. Egan J. B., 2005 Transcriptional interference–a crash course. Trends Genet. 21: 339–345. 15922833 10.1016/j.tig.2005.04.009 PMC2941638

[bib37] Shivaswamy S. Bhinge A. Zhao Y. Jones S. Hirst M., 2008 Dynamic remodeling of individual nucleosomes across a eukaryotic genome in response to transcriptional perturbation. PLoS Biol. 6: e65. 18351804 10.1371/journal.pbio.0060065 PMC2267817

[bib38] Sneppen K. Dodd I. B. Shearwin K. E. Palmer A. C. Schubert R. A., 2005 A mathematical model for transcriptional interference by RNA polymerase traffic in Escherichia coli. J. Mol. Biol. 346: 399–409. 15670592 10.1016/j.jmb.2004.11.075

[bib39] Terpe K., 2006 Overview of bacterial expression systems for heterologous protein production: from molecular and biochemical fundamentals to commercial systems. Appl. Microbiol. Biotechnol. 72: 211–222. 16791589 10.1007/s00253-006-0465-8

[bib40] Trinklein N. D. Aldred S. F. Hartman S. J. Schroeder D. I. Otillar R. P., 2004 An abundance of bidirectional promoters in the human genome. Genome Res. 14: 62–66. 14707170 10.1101/gr.1982804 PMC314279

[bib41] Wakano C. Byun J. S. Di L. J. Gardner K., 2012 The dual lives of bidirectional promoters. Biochim. Biophys. Acta 1819: 688–693. 22366276 10.1016/j.bbagrm.2012.02.006 PMC3371153

[bib42] Wang G. Z. Lercher M. J. Hurst L. D., 2011 Transcriptional coupling of neighboring genes and gene expression noise: evidence that gene orientation and noncoding transcripts are modulators of noise. Genome Biol. Evol. 3: 320–331. 21402863 10.1093/gbe/evr025 PMC5654408

[bib43] Woo Y. H. Li W. H., 2011 Gene clustering pattern, promoter architecture, and gene expression stability in eukaryotic genomes. Proc. Natl. Acad. Sci. USA 108: 3306–3311. 21300882 10.1073/pnas.1100210108 PMC3044413

[bib44] Xu Z. Wei W. Gagneur J. Perocchi F. Clauder-Munster S., 2009 Bidirectional promoters generate pervasive transcription in yeast. Nature 457: 1033–1037. 19169243 10.1038/nature07728 PMC2766638

