# Supplementary material for: Suppression of Expression Between Adjacent Genes Within Heterologous Modules in Yeast
Source: G3 (Bethesda). 2013 Nov 26;4(1):109–16. doi: 10.1534/g3.113.007922 (PMC3887525; doi:10.1534/g3.113.007922)
Supplement: Supporting Information [file supp_4_1_109__index.html]

Suppression of Expression Between Adjacent Genes Within Heterologous Modules in Yeast — Supporting Information 

# Suppression of Expression Between Adjacent Genes Within Heterologous Modules in Yeast

## Supporting Information for Lee *et al.*, 2014

**Files in this Data Supplement:**

- Supporting Information - Figures S1-S7 and Tables S1-S7 (PDF, 1 MB)
- Figure S1 - Variable growth on plates. (PDF, 450 KB)
- Figure S2 - Growth curves of the two control strains genomically integrated in opposite directions. (PDF, 359 KB)
- Figure S3 - Reproducible growth assay. (PDF, 393 KB)
- Figure S4 - GFP in Gal- and Gal+ conditions. (PDF, 377 KB)
- Figure S5 - Growth curves in Ura- and Ura+ conditions. (PDF, 398 KB)
- Figure S6 - Nucleosome mapping of the control strain in Gal- (dashed line) and GAL+ (continuous line) conditions. (PDF, 378 KB)
- Figure S7 - pGAL1 activation patterns. (PDF, 377 KB)
- Table S1 - Extracted growth rates for all strains. (PDF, 331 KB)
- Table S2 - Comparing the two control strains that differ only in the direction of genome integration. (PDF, 320 KB)
- Table S3 - Unique growth patterns in divergent strains. (PDF, 326 KB)
- Table S4 - KlURA3 transcript quantification in divergent strains. (PDF, 324 KB)
- Table S5 - Comparing the nucleosome occupancy between the divergent and control strains. (PDF, 423 KB)
- Table S6 - The plasmid map of our divergent heterologous module. (PDF, 310 KB)
- Table S7 - Primers used for nucleosome scanning assay. (PDF, 314 KB)
