# Supplementary material for: Suppression of Expression Between Adjacent Genes Within Heterologous Modules in Yeast
Source: G3 (Bethesda). 2013 Nov 26;4(1):109–16. doi: 10.1534/g3.113.007922 (PMC3887525; doi:10.1534/g3.113.007922)
Supplement: Supporting Information [file supp_g3.113.007922_007922SI.pdf]

G3 · Genes | Genomes | Genetics

Suppression of expression between adjacent genes within heterologous modules in yeast

Tae J. Lee[1,2], Rasesh Y. Parikh[2], Joshua S. Weitz[1,2] and Harold D. Kim[2]
[1]School of Biology and [2]School of Physics, Georgia Institute of Technology, Atlanta, Georgia, USA

DOI: 10.1534/g3.113.007922

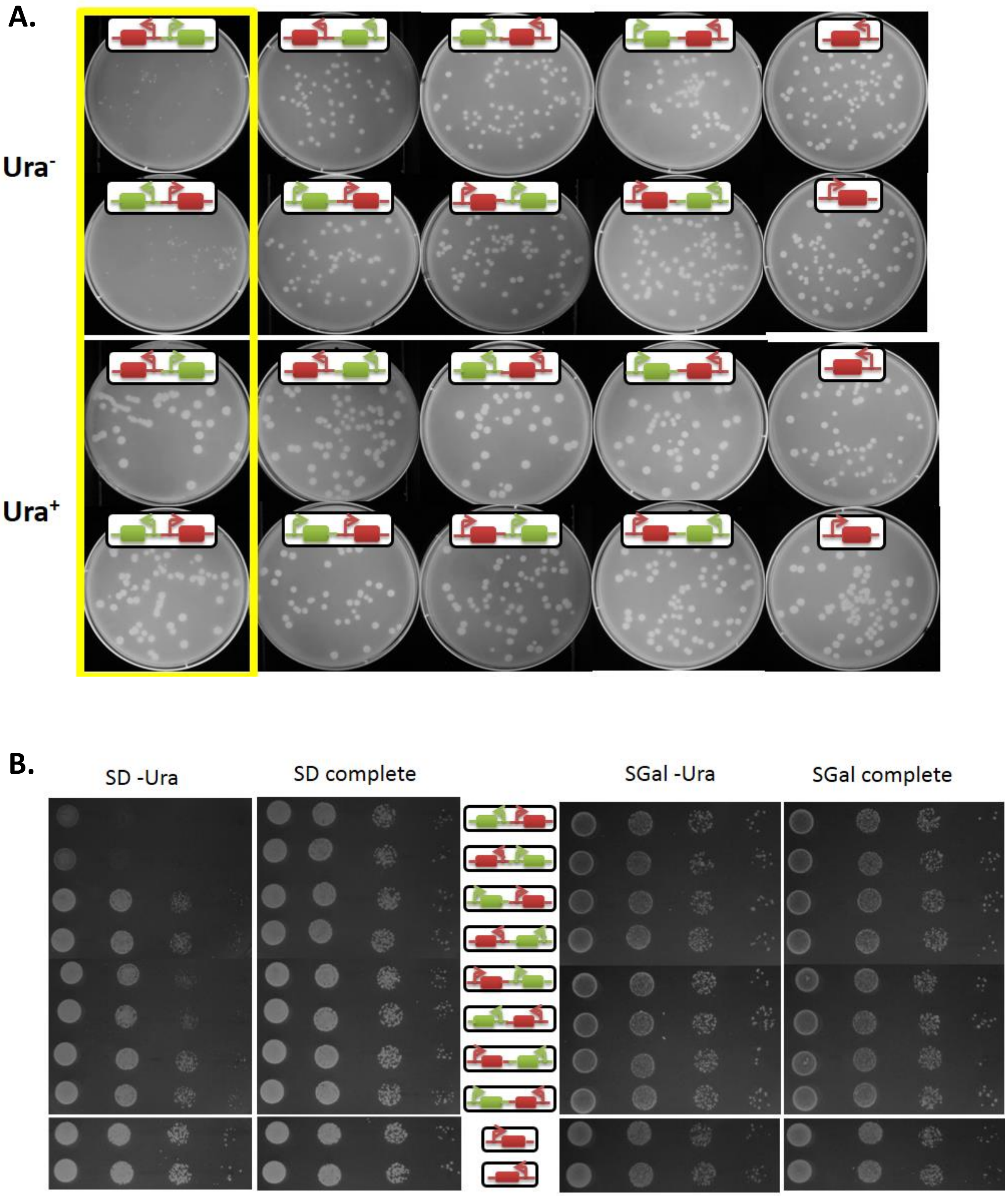

Figure S1 Variable growth on plates. A) Cells were grown for 2 days on glucose-supplemented SD agar plates in the absence (top plates) or presence of uracil (bottom plates). B) All strains were grown in liquid culture overnight then plated in serial dilution (with a dilution factor of 10) in 4 different conditions: SD-Ura vs. SD complete (left panels) and SGal-Ura vs. SGal complete (right panels). Prior to plating, all strains were concentrated at similar densities.

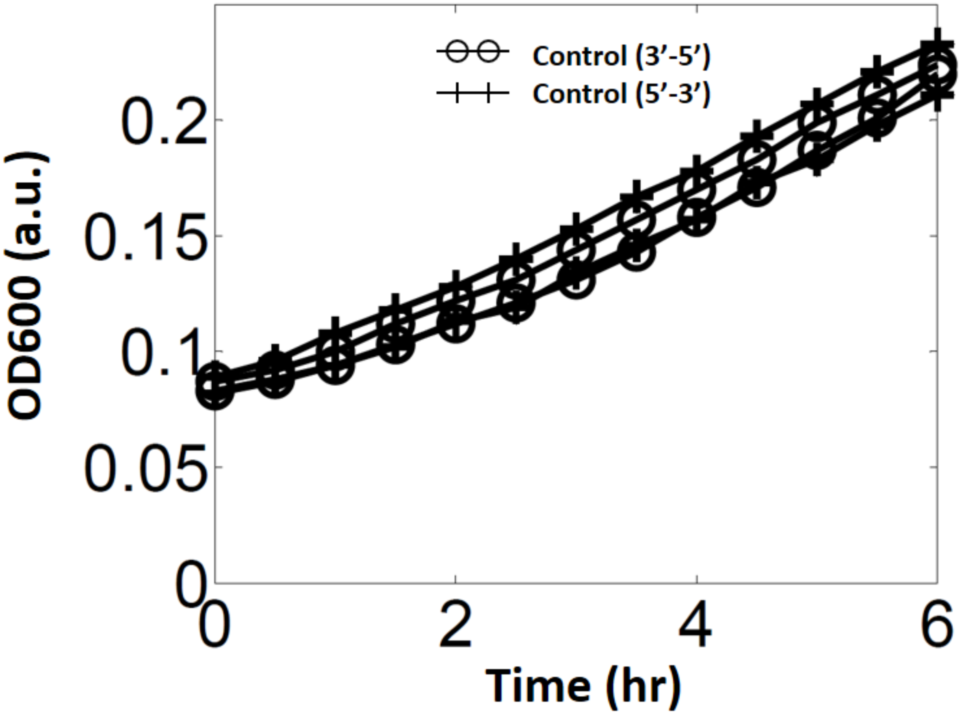

Figure S2 Growth curves of the two control strains genomically integrated in opposite directions. Control strains with the *KlURA3* gene integrated in 5'-3' ('represented by '+') and 3'-5' (represented by 'o') directions were grown on a 96-well plate at steady-state and their growth curves were measured with a plate reader. Two lines of the same marker shape represent two independent experiments conducted on different days with slightly different initial OD_{600}.

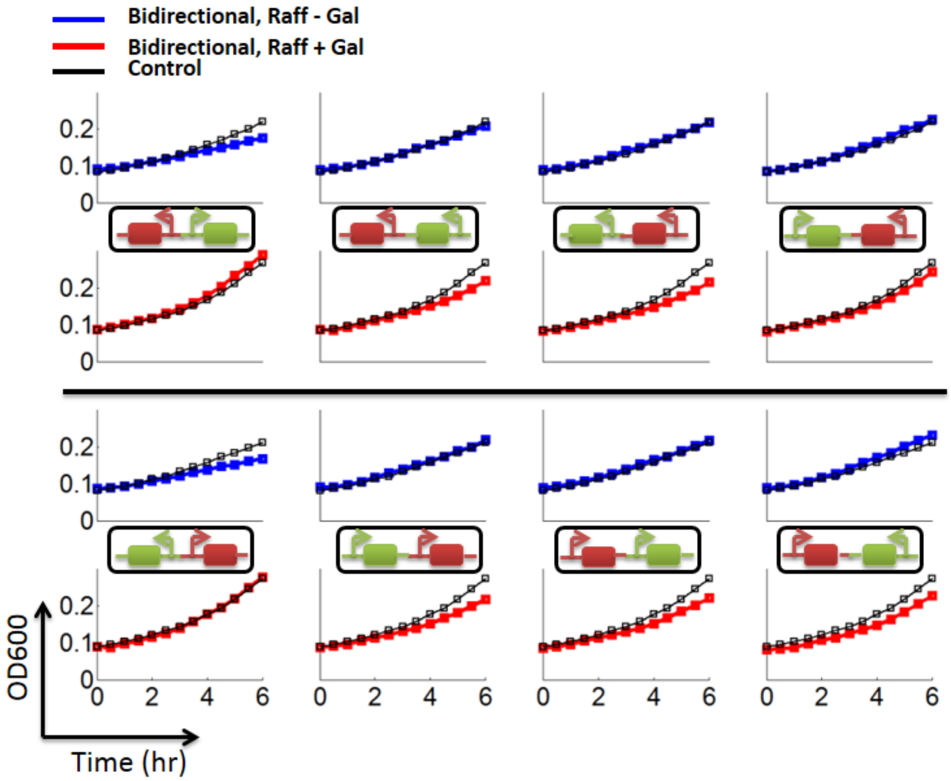

Figure S3 Reproducible growth assay. The steady-state growth curves in the Gal- (blue) and Gal+ (red) conditions are shown.

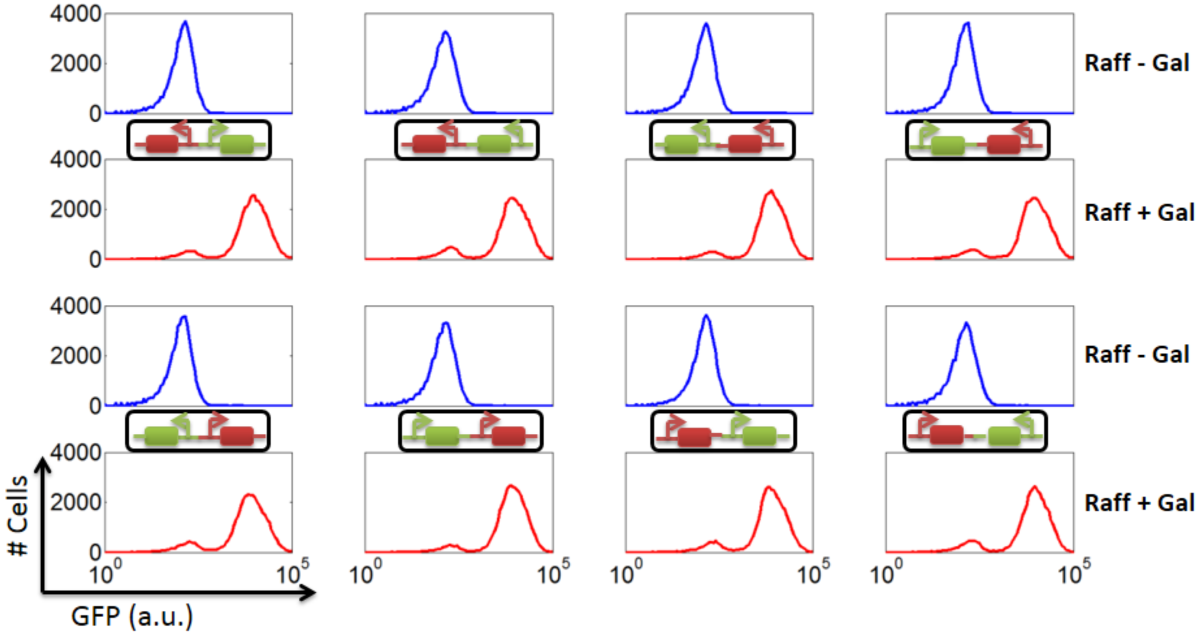

Figure S4 GFP in Gal$^-$ and Gal$^+$ conditions. After 24 hours of growth in either Gal$^-$ (blue histograms)) or Gal$^+$ (red histograms) condition, cells were washed, fixed, and measured for their GFP intensity with flow cytometry. The histograms are based on the GFP measurement of 50,000 cells.

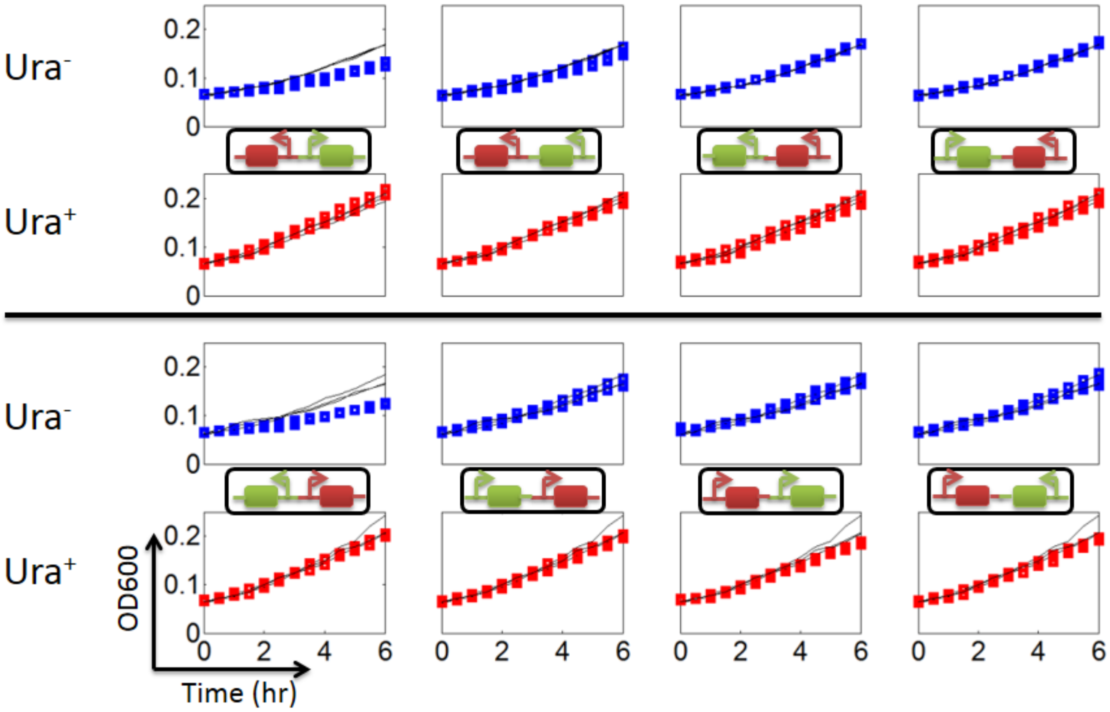

Figure S5 Growth curves in Ura$^-$ and Ura$^+$ conditions. After 24 hours of growth in either Ura$^-$ (blue lines) or Ura$^+$ (red lines) condition without galactose, cell growth at steady-state was measured for 6 hours at 30 min intervals with a plate reader. The growth curves of control strains are plotted in black for comparison. The different curves represent triplicate experiments.

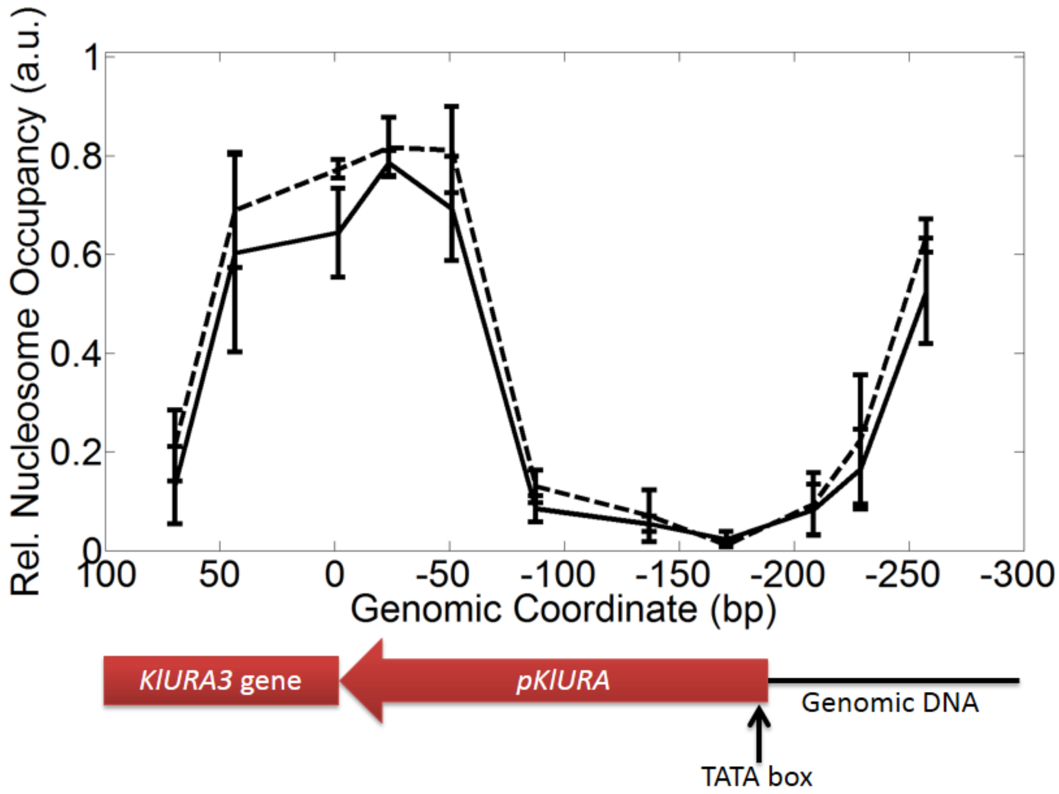

Figure S6 Nucleosome mapping of the control strain in Gal- (dashed line) and GAL+ (continuous line) conditions. The errorbars represent three independent measurements of relative nucleosome occupancy.

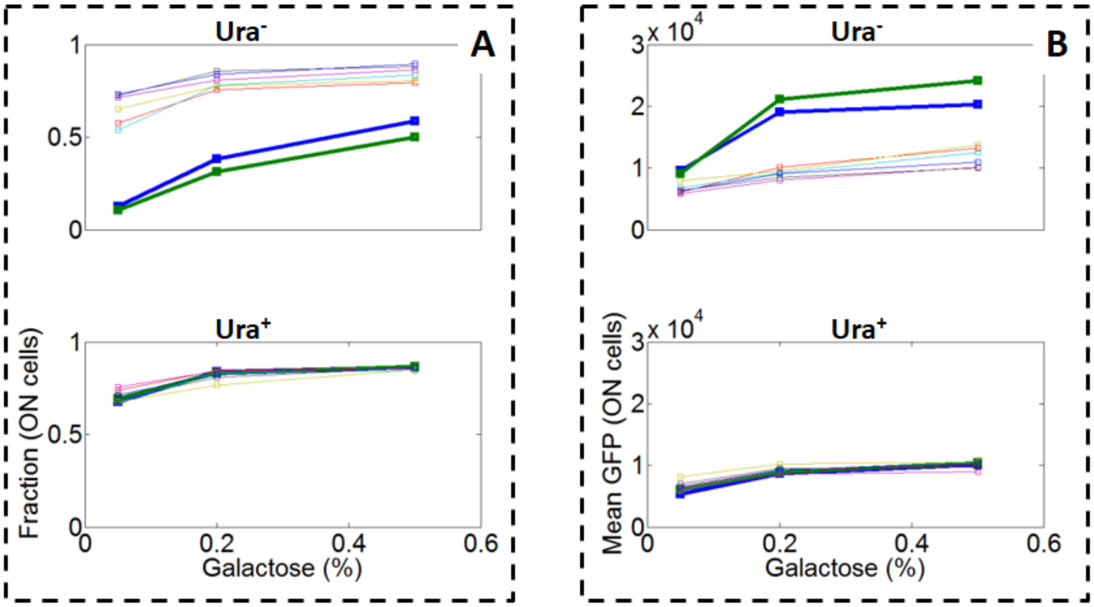

Figure S7 pGAL1 activation patterns. A) The fraction of ON cells in Ura- (top) and URA+ (bottom) conditions for increasing galactose concentrations (0.05, 0.2 and 0.5%). B) The mean GFP of the ON cells in Ura- (top) and Ura+ (bottom) conditions for increasing galactose concentrations (0.05, 0.2 and 0.5%). The bidirectional strains are plotted in thick green and blue lines, while the serial and convergent-promoter strains are plotted in thin lines.

Table S1 Extracted growth rates for all strains

		Divergent		Serial				Convergent		Control	
		←➔	⬅→	←⬅	➔→	⬅←	→➔	➔←	→⬅	←	→
Growth rates (hr^{-1}) in GAL-	Exp 1	0.117	0.117	0.153	0.155	0.158	0.157	0.174	0.175	0.167	0.165
	Exp 2	0.123	0.128	0.142	0.150	0.163	0.158	0.161	0.171	0.164	0.162
	Exp 3	0.130	0.115	0.192	0.196	0.205	0.191	0.199	0.198	0.211	0.212
	Exp 4	0.117	0.117	0.153	0.155	0.158	0.157	0.174	0.175	0.167	0.165
	Exp 5	0.132	0.129	0.161	0.165	0.176	0.177	0.182	0.189	0.179	0.197
	Avg±Std	0.12± 0.0069	0.12± 0.0065	0.16± 0.019	0.16± 0.019	0.17± 0.020	0.17± 0.016	0.18± 0.014	0.18± 0.012	0.18±0. 019	0.18± 0.023
Growth rates (hr^{-1}) in GAL+	Exp 1	0.213	0.209	0.160	0.156	0.156	0.162	0.180	0.180	0.200	0.192
	Exp 2	0.236	0.236	0.194	0.185	0.184	0.184	0.211	0.209	0.223	0.218
	Exp 3	0.241	0.237	0.152	0.165	0.150	0.155	0.198	0.203	0.204	0.184
	Exp 4	0.213	0.209	0.160	0.156	0.156	0.162	0.180	0.180	0.200	0.192
	Exp 5	0.234	0.233	0.140	0.140	0.159	0.141	0.158	N/A	0.168	0.171
	Avg±Std	0.23± 0.013	0.22± 0.015	0.16± 0.02	0.16± 0.016	0.16± 0.013	0.16± 0.016	0.19± 0.020	0.19± 0.015	0.20± 0.020	0.19± 0.017

The growth rates were obtained by fitting a line to a semi-log plot of the growth curves. For all growth rate extraction, the coefficient of determination (or R^2) was greater than 0.95. The mean and standard deviations represent at least three independent experiments. Thin and thick arrows denote the directionality of the KlURA3 gene and pGAL1-GFP, respectively.

Table S2   Comparing the two control strains that differ only in the direction of genome integration

	GAL-	GAL+
P-value	0.605	0.133
t-test statistic	-0.5755	2.05
Degrees of freedom	3	3

The statistical analysis is performed with paired t-tests to compare the growth rates between the two control strains (← and →) shown in Table S1. *T-tests with the p-value < 0.05 are assumed to show a statistically significant difference between the compared strains.

Table S3 Unique growth patterns in divergent strains

		Serial				Convergent		KlURA3 only	
		←⬅	➔→	⬅←	→➔	➔←	→⬅	←	→
GAL-	P-value	0.007*	0.0121*	0.0023*	0.004*	0.0004*	0.0052*	0.0015*	0.0055*
	T-test statistic	5.10	4.35	6.94	5.96	10.6	7.34	7.73	5.46
	Degrees of freedom	4	4	4	4	4	3	4	4
GAL+	P-value	0.0032*	0.0013*	0.0008*	0.0025*	0.0095*	0.0002*	0.0537	0.0276*
	T-test statistic	-6.30	-7.98	-9.18	-6.73	-4.67	-22.3	-2.71	-3.39
	Degrees of freedom	4	4	4	4	4	3	4	4

Thin and thick arrows denote the directionality of the KlURA3 gene and pGAL1-GFP, respectively. The statistical analysis is performed to illustrate the unique growth patterns in divergent strains compared to ‘other strains’ (serial, convergent, and control strains). To achieve this the growth rates of ‘other strains’ and divergent strains (shown in Table S1) are compared with paired t-tests. T-tests with statistically significant differences are marked with asterisks.

Table S4 KlURA3 transcript quantification in divergent strains

		←➔	🡸→
GAL-	Rel. transcript level	0.71±0.04	0.43±0.06
	P-value	0.0053	0.0035
	T-test statistic	-13.7	-16.8
	Degrees of freedom	2	2
GAL+	Rel. transcript level	2.4±0.31	2.04±0.19
	P-value	0.017	0.011
	T-test statistic	7.63	9.53
	Degrees of freedom	2	2

Thin and thick arrows denote the directionality of the KlURA gene and pGAL1-GFP, respectively. The transcript levels are normalized to those of control strains carrying the KlURA3 gene only. The mean and the standard deviation values are based on triplicate experiments. Paired t-tests comparing the relative KlURA3 transcript level in divergent strains with respect to '1' are shown.

Table S5   Comparing the nucleosome occupancy between the divergent and control strains

	Genomic coordinate (Control)	69	43	-1	-23	-51	-87	-137	**-170**	-208	-229	-257
	Genomic coordinate (Divergent)	69	43	-1	-23	-51	-87	-137	**-184**	-201	-236	-263
GAL-	p-value	0.30	0.17	0.61	0.65	0.056	0.058	*0.047**	***0.017****	*0.0086**	*0.0026**	0.58
	t-test statistic	-1.4	2.1	0.60	-0.52	-4.0	-4.0	*-4.4*	***-7.5***	*-11*	*-20*	-0.66
	Degrees of freedom	2	2	2	2	2	2	*2*	***2***	*2*	*2*	2
GAL+	p-value	0.34	0.39	0.16	0.34	0.39	0.36	0.057	**0.89**	0.56	0.82	*0.0062**
	t-test statistic	-1.2	1.1	2.2	1.3	1.1	-1.2	-4.0	**0.16**	0.69	-0.26	*13*
	Degrees of freedom	2	2	2	2	2	2	2	**2**	2	2	*2*

The DNA sequence for the first 7 genomic coordinates that span over pKlURA is identical between the divergent and control strains and the nucleosome occupancy at these positions were directly compared between them. The following coordinates span over either the genomic DNA (in control) or the pGAL1 promoter (in divergent). To perform statistical analysis, two nearest genomic coordinates on the divergent and control strains were paired and their nucleosome occupancy was compared with paired t-tests. T-tests with statistically significant differences are marked with asterisks. The genomic region over the putative TATA box are bolded.

Table S6 The plasmid map of our divergent heterologous module

GTGGATCTGATATCATCGATGAATTCAATGAAAGAGAGAGAGAGAAGCAAACAAAAAATTTTCAGTTCAATACAAC
AGATCACGTGATCTTTTGTAAGATGAAGTTGAAGTGAGTGTTGCACCGTGCCAATGCAGGTGGCTATTAGATTAA
ATATGTGATTTGTTCTATTAAGTTTCCTGTATAA[TTAATGGGGAGCGCTGATTCTCTTTTGGTACGCTTCCCATCC
AGCATTTCTGTATCTTTCACCTTCAACCTTAGGATCTCTACCCTTGGCGAAAAGTCCTCTGCCAACAATGATGATA
TCTGATCCACCACTTACAACTTCGTCGACGGTTCTGTACTGCTGACCCAATGCATCGCCTTTGTCGTCTAAACCT
ACACCTGGGGTCATGATTAGCCAATCAAACCCTTCTTCTCTTCCTCCCATATCGTTCTGAGCAATGAACCCAATAA
CGAAATCTTTATCACTCTTTGCAATATCAACGGTACCCTTAGTATATTCACCGTGTGCTAGAGAACCCTTGGAAGA
CAATTCAGCAAGCATCAATAATCCCCTTGGTTCTTTGGTGACCTCTTGCGCACCTTGTTTCAAGCCAGCAACAATA
CCAGCACCAGTAACCCCGTGGGCGTTGGTGATATCAGACCATTCTGCGATACGGTAAACGCCCGATGTATATTG
TAATTTGACTGTGTTACCGATATCGGCGAATTTTCTGTCCTCAAATATCAAGAACTTGTATTTCTCTGCCAATGCTT
TCAATGGAACGACAGTACCCTCATAACTGAAATCATCCAAGATATCAACGTGTGTTTTCAAAAGGCAAATGTATG
GACCCAACGTTTCAACAAGTTTCAATAGCTCATCAGTCGAACGAACGTCAAGAGAAGCACACAAATTGGTCTTCT
TTTCATCCATTAAACGTAAAAGTTTCGATGCAACCGGACTTGCATGAGTCTCAGCTCTACTGGTATATGATTTTGT
GGACAT][1][GGTGCAACTAATTGACGGGAGTGTATTGACGCTGGCGTACTGGCTTTCACAAAATGGCCCAATCAC
AACCACATCTTAGATAGTTGAAATGACTTTAGATAACATCAATTGAGATGAGCTTAATCATGTCAAAGCTAAAAGT
GTCACCATGAACGACAATTCTTAAGCAAATCACGTGATATAGATCC][2]AGATCTGGATCCAGATC{[TGTAAAGAG
CCCCATTATCTTAGCCTAAAAAAACCTTCTCTTTGGAACTTTCAGTAATACGCTTAACTGCTCATTGCTATATTGAA
GTACGGATTAGAAGCCGCCGAGCGGGTGACAGCCCTCCGAAGGAAGACTCTCCTCCGTGCGTCCTCGTCTTCA
CCGGTCGCGTTCCTGAAACGCAGATGTGCCTCGCGCCGCACTGCTCCGAACAATAAAGATTCTACAATACTAGC
TTTTATGGTTATGAAGAGGAAAAATTGGCAGTAACCTGGCCCCACAAACCTTCAAATGAACGAATCAAATTAACAA
CCATAGGATGATAATGCGATTAGTTTTTTAGCCTTATTTCTGGGGTAATTAATCAGCGAAGCGATGATTTTTGATC
TATTAACAGATATATAAATGCAAAAACTGCATAACCACTTTAACTAATACTTTCAACATTTTCGGTTTGTATTACTTC
TTATTCAAATGTAATAAAAGTATCAACAAAAAATTGTTAATATACCTCTATACTTTAACGTCAAGGAGAAAAAAC][3]C
CGGATCTCAAA[ATGTCTTTAATTAACAGTAAAGGAGAAGAACTTTTCACTGGAGTTGTCCCAATTCTTGTTGAATT
AGATGGTGATGTTAATGGGCACAAATTTTCTGTCAGTGGAGAGGGTGAAGGTGATGCAACATACGGAAAACTTA
CCCTTAAATTTATTTGCACTACTGGAAAACTACCTGTTCCATGGCCAACACTTGTCACTACTTTCACTTATGGTGTT
CAATGCTTTTCAAGATACCCAGATCATATGAAACGGCATGACTTTTTCAAGAGTGCCATGCCCGAAGGTTATGTA
CAGGAAAGAACTATATTTTTCAAAGATGACGGGAACTACAAGACACGTGCTGAAGTCAAGTTTGAAGGTGATACC
CTTGTTAATAGAATCGAGTTAAAAGGTATTGATTTTAAAGAAGATGGAAACATTCTTGGACACAAATTGGAATACA
ACTATAACTCACACAATGTATACATCATGGCAGACAAACAAAAGAATGGAATCAAAGTTAACTTCAAAATTAGACA
CAACATTGAAGATGGAAGCGTTCAACTAGCAGACCATTATCAACAAAATACTCCAATTGGCGATGGCCCTGTCCT
TTTACCAGACAACCATTACCTGTCCACACAATCTGCCCTTTCGAAAGATCCCAACGAAAAGAGAGACCACATGGT
CCTTCTTGAGTTTGTAACAGCTGCTGGGATTACACATGGCATGGATGAACTATACAAATAGGGCGCGCC][4]ACTT
CTAAATAA[GCGAATTTCTTATGATTTATGATTTTTATTATTAAATAAGTTATAAAAAAAATAAGTGTATACAAATTTT
AAAGTGACTCTTAGGTTTTAAAACGAAAATTCTTATTCTTGAGTAACTCTTTCCTGTAGGTCAGGTTGCTTTCTCAG
GTATAGTATGAGGTCGCTCTTATTGACCACACC][5]GAATTCGGATCCGTCGACCTGCAGCGT}[6]

[ ][1]: KlURA3 ORF; [ ][2]: pKlURA3 promoter; [ ][3]: pGAL1 promoter; [ ][4]: GFP(S65) ORF; [ ][5]: yADH1 terminator; { }[6]: pGAL1-GFP (with yADH terminator) from [2]; the underlined region denotes the match between the plasmid map and sequenced results.

Table S7 Primers used for nucleosome scanning assay

Primer set	Control strain	divergent strain
1	FW1: CACACAAATTGGTCTTCTTTTCATCC	Same as FW1
	RV1:CAAGTCCGGTTGCATCGAAAC	Same as RV1
2	FW2: CGTAAAAGTTTCGATGCAACC	Same as FW2
	RV2: CAGTAGAGCTGAGACTCATGCAA	Same as RV2
3	FW3: GCTCTACTGGTATATGATTTTGTGG	Same as FW3
	RV3: CGTCAATACACTCCCGTCAA	Same as RV3
4	FW4: TTTGTGGACATGGTGCAACT	Same as FW4
	RV4: GCCATTTTGTGAAAGCCAGT	Same as RV4
5	FW5: GTATTGACGCTGGCGTACTG	Same as FW5
	RV5: TCTAAGATGTGGTTGTGATTGG	Same as RV5
6	FW6: GGCCCAATCACAACCACATC	Same as FW6
	RV6:GCTCATCTCAATTGATGTTATCTAAAGTC	Same as RV6
7	FW7: CAATTGAGATGAGCTTAATCATGTCAAAGC	Same as FW7
	RV7: GCTTAAGAATTGTCGTTCATGG	Same as RV7
8	FW8: GAACGACAATTCTTAAGCAAATCACGTG	Same as FW8
	RV8: CGACCTTCCATTGGATCTATATCACGT	RV8': GGGGCTCTTTACAGATCTGGATCC
9	FW9: CGTGATATAGATCCAATGGAAGG	FW9': CTTAAGCAAATCACGTGATATAGATCC
	RV9: GAGTTCTGTATTGTTCTTCTTAGTGC	RV9': GGTTTTTTTAGGCTAAGATAATGGGGC
10	FW10: CGGGATGAGCATATACAAGC	FW10': AAAGAGCCCCATTATCTTAGCC
	RV10: GCACAATAATACCGTGTAGAG	RV10': ATTACTGAAAGTTCCAAAGAGAAGG
11	FW11: GAACAATACAGAACTCTACACGG	FW11': CCTTCTCTTTGGAACTTTCAGT
	RV11: AAAACACTCGGTTTTACTCGAGC	RV11': AATATAGCAATGAGCAGTTAAGCG
Reference*	FWR: CCTTTAGCTAATAGAGTAAGCCACA	
	RVR: TTTAACACTACTGGTTTATGAAAGAAA	

*The reference primers target a constitutively nucleosomal region that is regulated independently of galactose and uracil, and thus can serve as a proper normalization standard [1].

Reference:

1. Kim HD, O'Shea EK (2008) A quantitative model of transcription factor-activated gene expression. Nat Struct Mol Biol 15: 1192-1198.
2. Longtine MS, McKenzie A, 3rd, Demarini DJ, Shah NG, Wach A, et al. (1998) Additional modules for versatile and economical PCR-based gene deletion and modification in Saccharomyces cerevisiae. Yeast 14: 953-961.
