# Supplementary material for: Suppression of Expression Between Adjacent Genes Within Heterologous Modules in Yeast
Source: G3 (Bethesda). 2013 Nov 26;4(1):109–16. doi: 10.1534/g3.113.007922 (PMC3887525; doi:10.1534/g3.113.007922)
Supplement: Supporting Information [file supp_g3.113.007922_FigureS1.pdf]

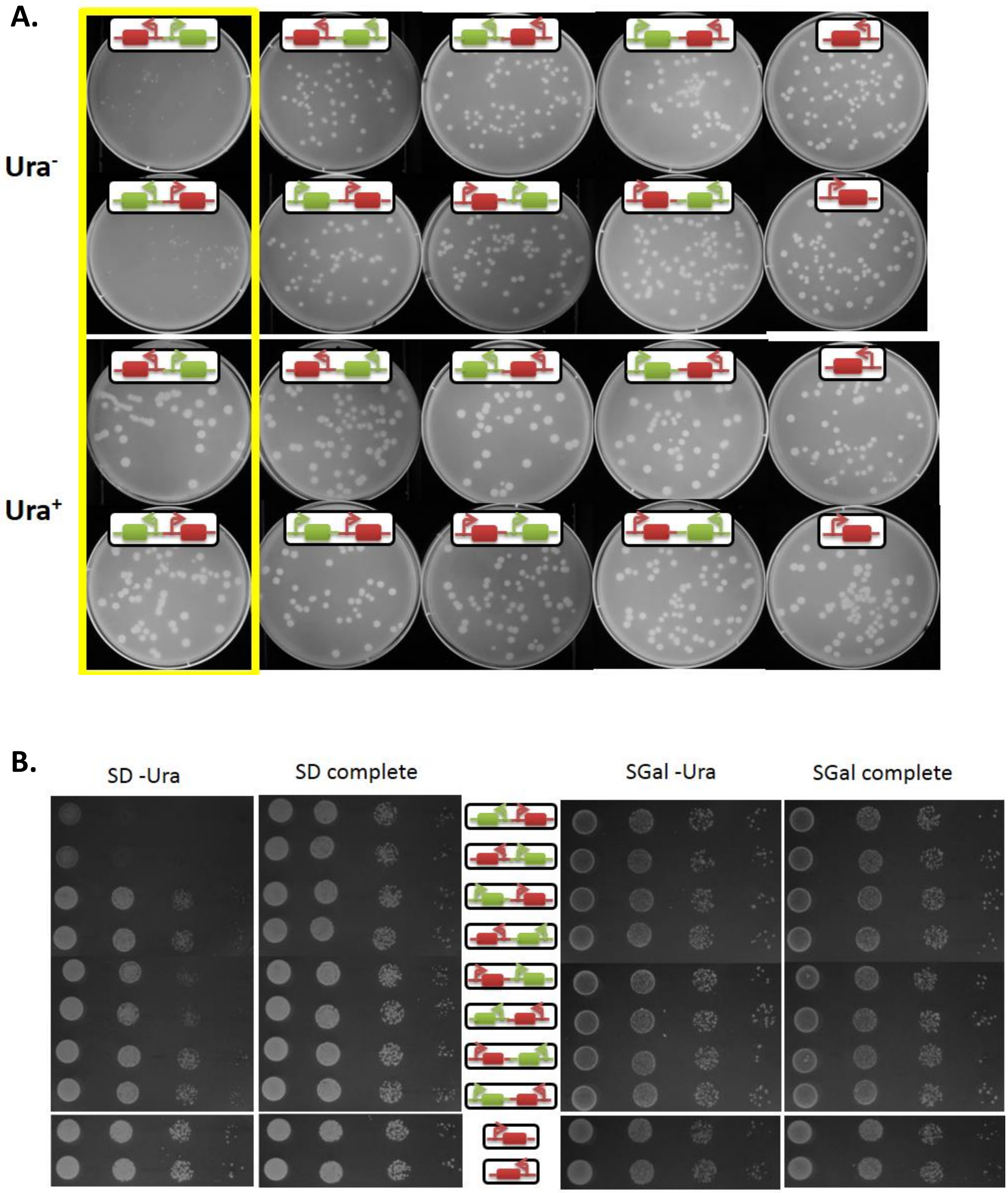

Figure S1 Variable growth on plates. A) Cells were grown for 2 days on glucose-supplemented SD agar plates in the absence (top plates) or presence of uracil (bottom plates). B) All strains were grown in liquid culture overnight then plated in serial dilution (with a dilution factor of 10) in 4 different conditions: SD-Ura vs. SD complete (left panels) and SGal-Ura vs. SGal complete (right panels). Prior to plating, all strains were concentrated at similar densities.
