# Supplementary material for: Suppression of Expression Between Adjacent Genes Within Heterologous Modules in Yeast
Source: G3 (Bethesda). 2013 Nov 26;4(1):109–16. doi: 10.1534/g3.113.007922 (PMC3887525; doi:10.1534/g3.113.007922)
Supplement: Supporting Information [file supp_g3.113.007922_FigureS2.pdf]

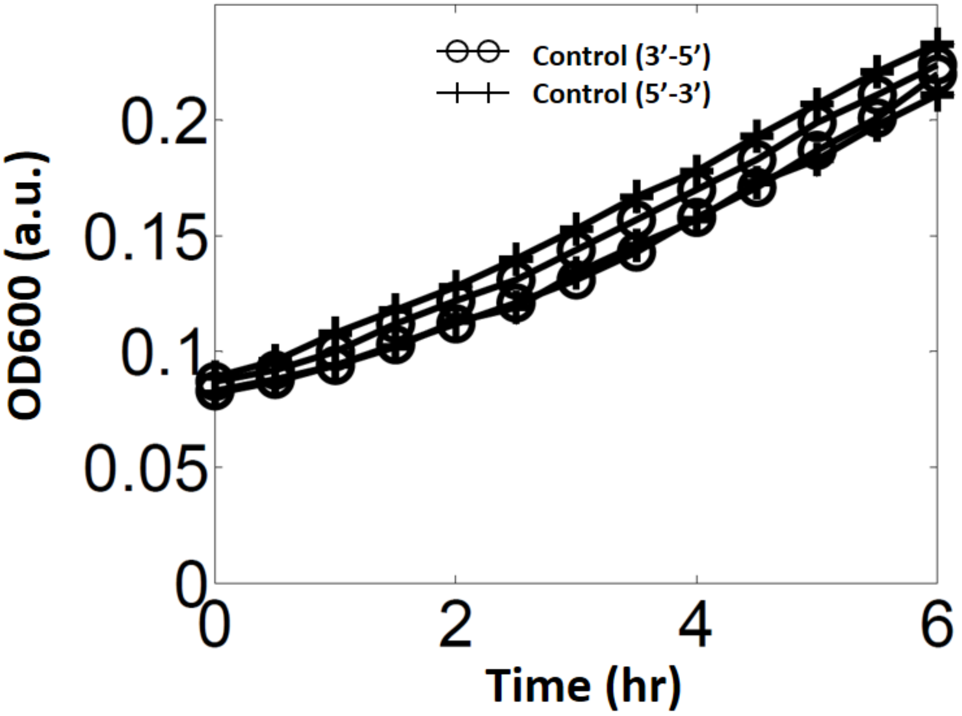

Figure S2 Growth curves of the two control strains genomically integrated in opposite directions. Control strains with the *KlURA3* gene integrated in 5'-3' ('represented by '+') and 3'-5' (represented by 'o') directions were grown on a 96-well plate at steady-state and their growth curves were measured with a plate reader. Two lines of the same marker shape represent two independent experiments conducted on different days with slightly different initial OD_{600}.
