# Supplementary material for: Suppression of Expression Between Adjacent Genes Within Heterologous Modules in Yeast
Source: G3 (Bethesda). 2013 Nov 26;4(1):109–16. doi: 10.1534/g3.113.007922 (PMC3887525; doi:10.1534/g3.113.007922)
Supplement: Supporting Information [file supp_g3.113.007922_FigureS3.pdf]

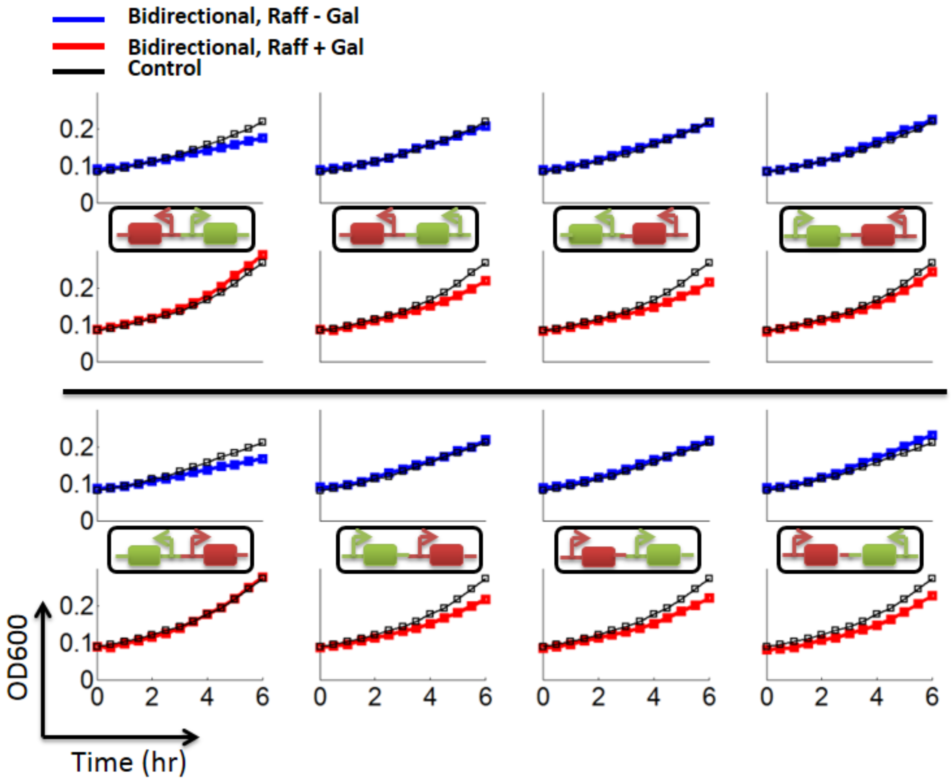

Figure S3 Reproducible growth assay. The steady-state growth curves in the Gal- (blue) and Gal+ (red) conditions are shown.
