# Supplementary material for: Suppression of Expression Between Adjacent Genes Within Heterologous Modules in Yeast
Source: G3 (Bethesda). 2013 Nov 26;4(1):109–16. doi: 10.1534/g3.113.007922 (PMC3887525; doi:10.1534/g3.113.007922)
Supplement: Supporting Information [file supp_g3.113.007922_FigureS4.pdf]

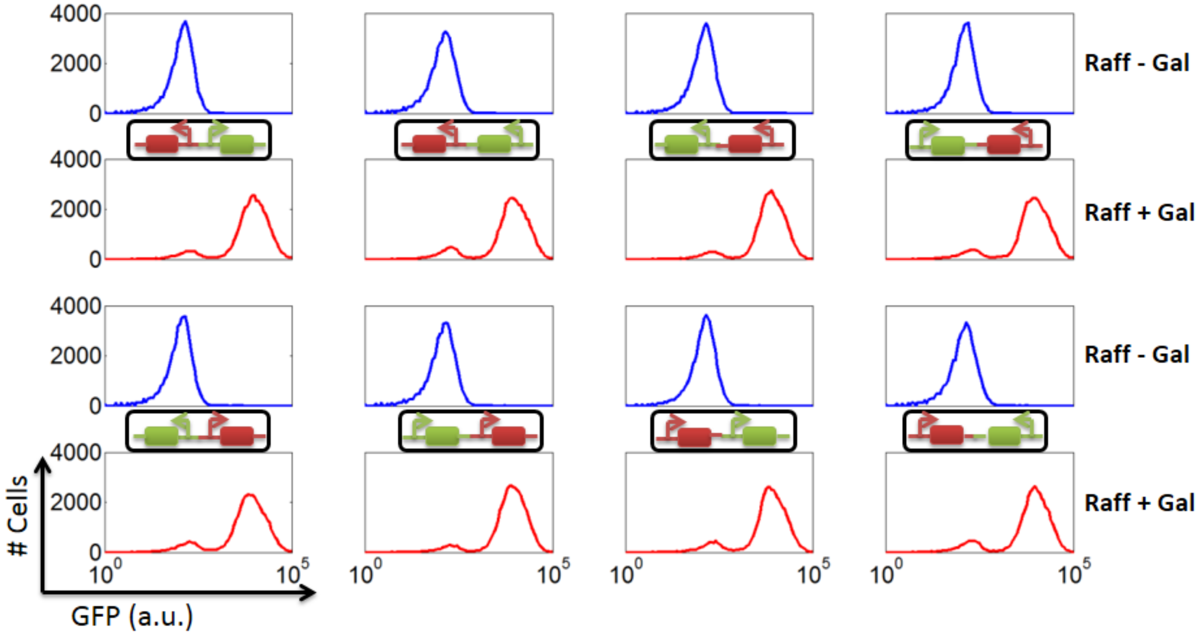

Figure S4 GFP in Gal$^-$ and Gal$^+$ conditions. After 24 hours of growth in either Gal$^-$ (blue histograms)) or Gal$^+$ (red histograms) condition, cells were washed, fixed, and measured for their GFP intensity with flow cytometry. The histograms are based on the GFP measurement of 50,000 cells.
