# Supplementary material for: Suppression of Expression Between Adjacent Genes Within Heterologous Modules in Yeast
Source: G3 (Bethesda). 2013 Nov 26;4(1):109–16. doi: 10.1534/g3.113.007922 (PMC3887525; doi:10.1534/g3.113.007922)
Supplement: Supporting Information [file supp_g3.113.007922_FigureS5.pdf]

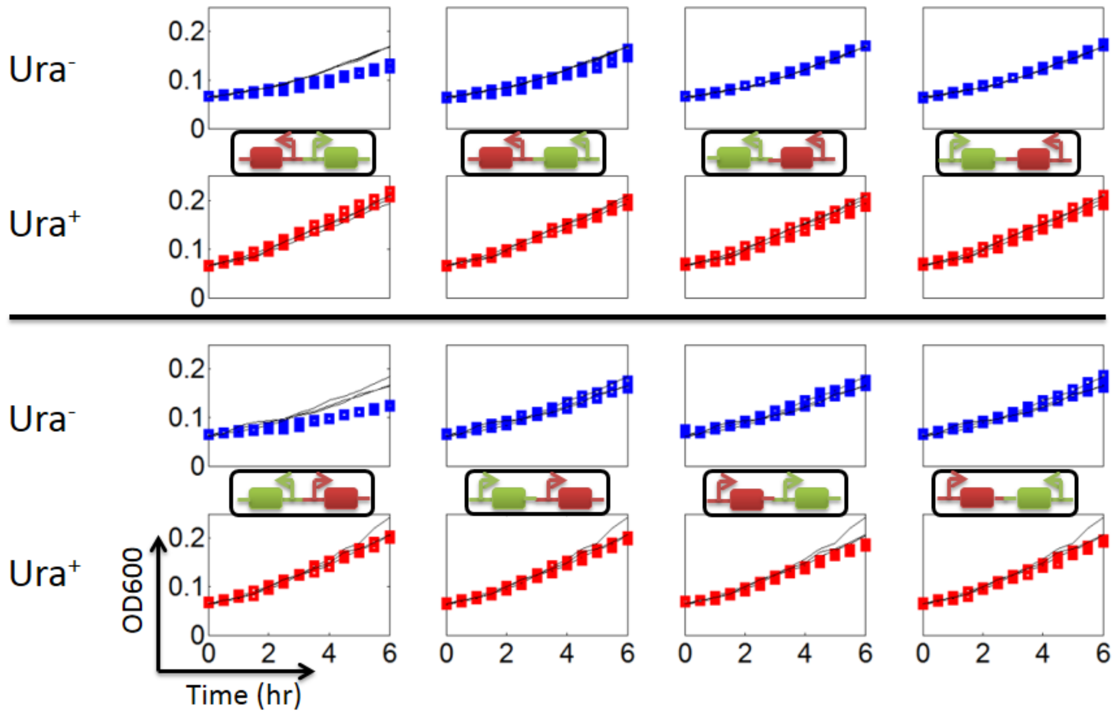

Figure S5 Growth curves in Ura^- and Ura^+ conditions. After 24 hours of growth in either Ura^- (blue lines) or Ura^+ (red lines) condition without galactose, cell growth at steady-state was measured for 6 hours at 30 min intervals with a plate reader. The growth curves of control strains are plotted in black for comparison. The different curves represent triplicate experiments.
