# Supplementary material for: Suppression of Expression Between Adjacent Genes Within Heterologous Modules in Yeast
Source: G3 (Bethesda). 2013 Nov 26;4(1):109–16. doi: 10.1534/g3.113.007922 (PMC3887525; doi:10.1534/g3.113.007922)
Supplement: Supporting Information [file supp_g3.113.007922_FigureS7.pdf]

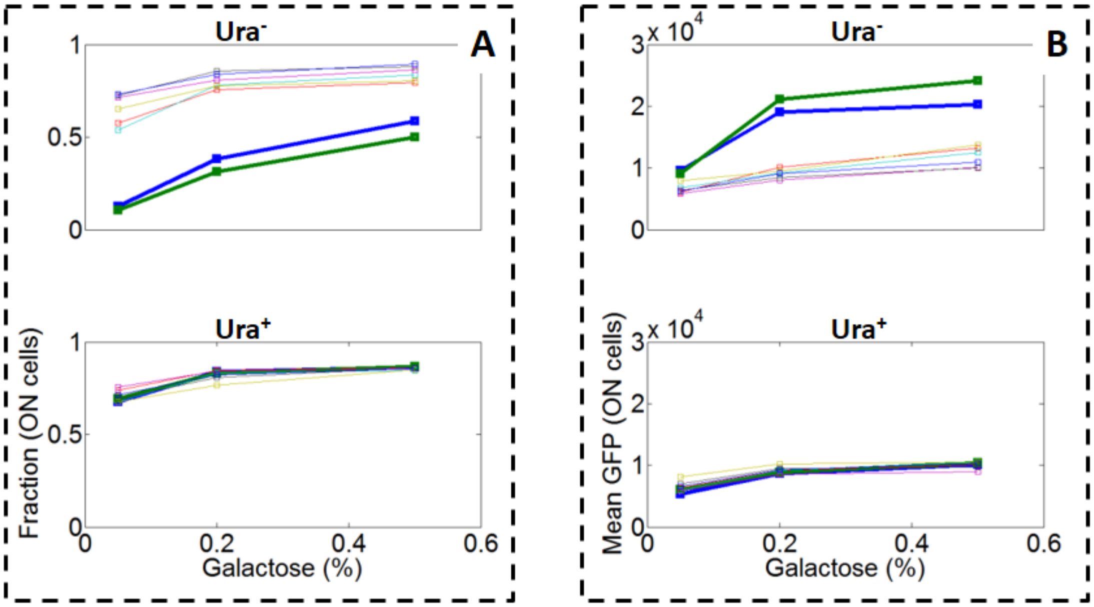

Figure S7 pGAL1 activation patterns. A) The fraction of ON cells in Ura$^-$ (top) and URA+ (bottom) conditions for increasing galactose concentrations (0.05, 0.2 and 0.5%). B) The mean GFP of the ON cells in Ura$^-$ (top) and Ura$^+$ (bottom) conditions for increasing galactose concentrations (0.05, 0.2 and 0.5%). The bidirectional strains are plotted in thick green and blue lines, while the serial and convergent-promoter strains are plotted in thin lines.
