# Supplementary material for: Suppression of Expression Between Adjacent Genes Within Heterologous Modules in Yeast
Source: G3 (Bethesda). 2013 Nov 26;4(1):109–16. doi: 10.1534/g3.113.007922 (PMC3887525; doi:10.1534/g3.113.007922)
Supplement: Supporting Information [file supp_g3.113.007922_TableS1.pdf]

Table S1 Extracted growth rates for all strains

		Divergent		Serial				Convergent		Control	
		←➔	⬅→	←⬅	➔→	⬅←	→➔	➔←	→⬅	←	→
Growth rates (hr^{-1}) in GAL-	Exp 1	0.117	0.117	0.153	0.155	0.158	0.157	0.174	0.175	0.167	0.165
	Exp 2	0.123	0.128	0.142	0.150	0.163	0.158	0.161	0.171	0.164	0.162
	Exp 3	0.130	0.115	0.192	0.196	0.205	0.191	0.199	0.198	0.211	0.212
	Exp 4	0.117	0.117	0.153	0.155	0.158	0.157	0.174	0.175	0.167	0.165
	Exp 5	0.132	0.129	0.161	0.165	0.176	0.177	0.182	0.189	0.179	0.197
	Avg±Std	0.12± 0.0069	0.12± 0.0065	0.16± 0.019	0.16± 0.019	0.17± 0.020	0.17± 0.016	0.18± 0.014	0.18± 0.012	0.18±0.019	0.18± 0.023
Growth rates (hr^{-1}) in GAL+	Exp 1	0.213	0.209	0.160	0.156	0.156	0.162	0.180	0.180	0.200	0.192
	Exp 2	0.236	0.236	0.194	0.185	0.184	0.184	0.211	0.209	0.223	0.218
	Exp 3	0.241	0.237	0.152	0.165	0.150	0.155	0.198	0.203	0.204	0.184
	Exp 4	0.213	0.209	0.160	0.156	0.156	0.162	0.180	0.180	0.200	0.192
	Exp 5	0.234	0.233	0.140	0.140	0.159	0.141	0.158	N/A	0.168	0.171
	Avg±Std	0.23± 0.013	0.22± 0.015	0.16± 0.02	0.16± 0.016	0.16± 0.013	0.16± 0.016	0.19± 0.020	0.19± 0.015	0.20± 0.020	0.19± 0.017

The growth rates were obtained by fitting a line to a semi-log plot of the growth curves. For all growth rate extraction, the coefficient of determination (or R^2) was greater than 0.95. The mean and standard deviations represent at least three independent experiments. Thin and thick arrows denote the directionality of the KlURA3 gene and pGAL1-GFP, respectively.
