# Supplementary material for: Suppression of Expression Between Adjacent Genes Within Heterologous Modules in Yeast
Source: G3 (Bethesda). 2013 Nov 26;4(1):109–16. doi: 10.1534/g3.113.007922 (PMC3887525; doi:10.1534/g3.113.007922)
Supplement: Supporting Information [file supp_g3.113.007922_TableS2.pdf]

Table S2 Comparing the two control strains that differ only in the direction of genome integration

	GAL-	GAL+
P-value	0.605	0.133
t-test statistic	-0.5755	2.05
Degrees of freedom	3	3

The statistical analysis is performed with paired t-tests to compare the growth rates between the two control strains (← and →) shown in Table S1. *T-tests with the p-value < 0.05 are assumed to show a statistically significant difference between the compared strains.
