# Supplementary material for: Suppression of Expression Between Adjacent Genes Within Heterologous Modules in Yeast
Source: G3 (Bethesda). 2013 Nov 26;4(1):109–16. doi: 10.1534/g3.113.007922 (PMC3887525; doi:10.1534/g3.113.007922)
Supplement: Supporting Information [file supp_g3.113.007922_TableS3.pdf]

**Table S3 Unique growth patterns in divergent strains**

| | | Serial | | | | Convergent | | KlURA3 only | |
|---|---|---|---|---|---|---|---|---|---|
| | | ←⬅ | ➡→ | ⬅← | →➡ | ➡← | →⬅ | ← | → |
| GAL- | P-value | *0.007** | *0.0121** | *0.0023** | *0.004** | *0.0004** | *0.0052** | *0.0015** | *0.0055** |
| | T-test statistic | *5.10* | *4.35* | *6.94* | *5.96* | *10.6* | *7.34* | *7.73* | *5.46* |
| | Degrees of freedom | *4* | *4* | *4* | *4* | *4* | *3* | *4* | *4* |
| GAL+ | P-value | *0.0032** | *0.0013** | *0.0008** | *0.0025** | *0.0095** | *0.0002** | 0.0537 | *0.0276** |
| | T-test statistic | *-6.30* | *-7.98* | *-9.18* | *-6.73* | *-4.67* | *-22.3* | *-2.71* | *-3.39* |
| | Degrees of freedom | *4* | *4* | *4* | *4* | *4* | *3* | *4* | *4* |

Thin and thick arrows denote the directionality of the KlURA3 gene and pGAL1-GFP, respectively. The statistical analysis is performed to illustrate the unique growth patterns in divergent strains compared to 'other strains' (serial, convergent, and control strains). To achieve this the growth rates of 'other strains' and divergent strains (shown in Table S1) are compared with paired t-tests. T-tests with statistically significant differences are marked with asterisks.
