# Supplementary material for: Suppression of Expression Between Adjacent Genes Within Heterologous Modules in Yeast
Source: G3 (Bethesda). 2013 Nov 26;4(1):109–16. doi: 10.1534/g3.113.007922 (PMC3887525; doi:10.1534/g3.113.007922)
Supplement: Supporting Information [file supp_g3.113.007922_TableS4.pdf]

Table S4   KlURA3 transcript quantification in divergent strains

		←**➔**	**🡐**→
GAL-	Rel. transcript level	0.71±0.04	0.43±0.06
	P-value	0.0053	0.0035
	T-test statistic	-13.7	-16.8
	Degrees of freedom	2	2
GAL+	Rel. transcript level	2.4±0.31	2.04±0.19
	P-value	0.017	0.011
	T-test statistic	7.63	9.53
	Degrees of freedom	2	2

Thin and thick arrows denote the directionality of the KlURA gene and pGAL1-GFP, respectively. The transcript levels are normalized to those of control strains carrying the KlURA3 gene only. The mean and the standard deviation values are based on triplicate experiments. Paired t-tests comparing the relative KlURA3 transcript level in divergent strains with respect to '1' are shown.
