# Supplementary material for: Suppression of Expression Between Adjacent Genes Within Heterologous Modules in Yeast
Source: G3 (Bethesda). 2013 Nov 26;4(1):109–16. doi: 10.1534/g3.113.007922 (PMC3887525; doi:10.1534/g3.113.007922)
Supplement: Supporting Information [file supp_g3.113.007922_TableS5.pdf]

Table S5 Comparing the nucleosome occupancy between the divergent and control strains

	Genomic coordinate (Control)	69	43	-1	-23	-51	-87	-137	**-170**	-208	-229	-257
	Genomic coordinate (Divergent)	69	43	-1	-23	-51	-87	-137	**-184**	-201	-236	-263
GAL-	p-value	0.30	0.17	0.61	0.65	0.056	0.058	*0.047**	***0.017****	*0.0086**	*0.0026**	0.58
	t-test statistic	-1.4	2.1	0.60	-0.52	-4.0	-4.0	*-4.4*	***-7.5***	*-11*	*-20*	-0.66
	Degrees of freedom	2	2	2	2	2	2	*2*	***2***	*2*	*2*	2
GAL+	p-value	0.34	0.39	0.16	0.34	0.39	0.36	0.057	**0.89**	0.56	0.82	*0.0062**
	t-test statistic	-1.2	1.1	2.2	1.3	1.1	-1.2	-4.0	**0.16**	0.69	-0.26	*13*
	Degrees of freedom	2	2	2	2	2	2	2	**2**	2	2	*2*

The DNA sequence for the first 7 genomic coordinates that span over pKlURA is identical between the divergent and control strains and the nucleosome occupancy at these positions were directly compared between them. The following coordinates span over either the genomic DNA (in control) or the pGAL1 promoter (in divergent). To perform statistical analysis, two nearest genomic coordinates on the divergent and control strains were paired and their nucleosome occupancy was compared with paired t-tests. T-tests with statistically significant differences are marked with asterisks. The genomic region over the putative TATA box are bolded.
