# Supplementary material for: Suppression of Expression Between Adjacent Genes Within Heterologous Modules in Yeast
Source: G3 (Bethesda). 2013 Nov 26;4(1):109–16. doi: 10.1534/g3.113.007922 (PMC3887525; doi:10.1534/g3.113.007922)
Supplement: Supporting Information [file supp_g3.113.007922_TableS6.pdf]

**Table S6 The plasmid map of our divergent heterologous module**

GTGGATCTGATATCATCGATGAATTCAATGAAAGAGAGAGAGAGAAGCAAACAAAAAATTTTCAGTTCAATACAAC
AGATCACGTGATCTTTTGTAAGATGAAGTTGAAGTGAGTGTTGCACCGTGCCAATGCAGGTGGCTATTAGATTAA
ATATGTGATTTGTTCTATTAAGTTTCCTGTATAA[TTAATGGGGAGCGCTGATTCTCTTTTGGTACGCTTCCCATCC
AGCATTTCTGTATCTTTCACCTTCAACCTTAGGATCTCTACCCTTGGCGAAAAGTCCTCTGCCAACAATGATGATA
TCTGATCCACCACTTACAACTTCGTCGACGGTTCTGTACTGCTGACCCAATGCATCGCCTTTGTCGTCTAAACCT
ACACCTGGGGTCATGATTAGCCAATCAAACCCTTCTTCTCTTCCTCCCATATCGTTCTGAGCAATGAACCCAATAA
CGAAATCTTTATCACTCTTTGCAATATCAACGGTACCCTTAGTATATTCACCGTGTGCTAGAGAACCCTTGGAAGA
CAATTCAGCAAGCATCAATAATCCCCTTGGTTCTTTGGTGACCTCTTGCGCACCTTGTTTCAAGCCAGCAACAATA
CCAGCACCAGTAACCCCGTGGGCGTTGGTGATATCAGACCATTCTGCGATACGGTAAACGCCCGATGTATATTG
TAATTTGACTGTGTTACCGATATCGGCGAATTTTCTGTCCTCAAATATCAAGAACTTGTATTTCTCTGCCAATGCTT
TCAATGGAACGACAGTACCCTCATAACTGAAATCATCCAAGATATCAACGTGTGTTTTCAAAAGGCAAATGTATG
GACCCAACGTTTCAACAAGTTTCAATAGCTCATCAGTCGAACGAACGTCAAGAGAAGCACACAAATTGGTCTTCT
TTTCATCCATTAAACGTAAAAGTTTCGATGCAACCGGACTTGCATGAGTCTCAGCTCTACTGGTATATGATTTTGT
GGACAT][1][GGTGCAACTAATTGACGGGAGTGTATTGACGCTGGCGTACTGGCTTTCACAAAATGGCCCAATCAC
AACCACATCTTAGATAGTTGAAATGACTTTAGATAACATCAATTGAGATGAGCTTAATCATGTCAAAGCTAAAAGT
GTCACCATGAACGACAATTCTTAAGCAAATCACGTGATATAGATCC][2]AGATCTGGATCCAGATC{[TGTAAAGAG
CCCCATTATCTTAGCCTAAAAAAACCTTCTCTTTGGAACTTTCAGTAATACGCTTAACTGCTCATTGCTATATTGAA
GTACGGATTAGAAGCCGCCGAGCGGGTGACAGCCCTCCGAAGGAAGACTCTCCTCCGTGCGTCCTCGTCTTCA
CCGGTCGCGTTCCTGAAACGCAGATGTGCCTCGCGCCGCACTGCTCCGAACAATAAAGATTCTACAATACTAGC
TTTTATGGTTATGAAGAGGAAAAATTGGCAGTAACCTGGCCCCACAAACCTTCAAATGAACGAATCAAATTAACAA
CCATAGGATGATAATGCGATTAGTTTTTTAGCCTTATTTCTGGGGTAATTAATCAGCGAAGCGATGATTTTTGATC
TATTAACAGATATATAAATGCAAAAACTGCATAACCACTTTAACTAATACTTTCAACATTTTCGGTTTGTATTACTTC
TTATTCAAATGTAATAAAAGTATCAACAAAAAATTGTTAATATACCTCTATACTTTAACGTCAAGGAGAAAAAAC][3]C
CGGATCTCAAA[ATGTCTTTAATTAACAGTAAAGGAGAAGAACTTTTCACTGGAGTTGTCCCAATTCTTGTTGAATT
AGATGGTGATGTTAATGGGCACAAATTTTCTGTCAGTGGAGAGGGTGAAGGTGATGCAACATACGGAAAACTTA
CCCTTAAATTTATTTGCACTACTGGAAAACTACCTGTTCCATGGCCAACACTTGTCACTACTTTCACTTATGGTGTT
CAATGCTTTTCAAGATACCCAGATCATATGAAACGGCATGACTTTTTCAAGAGTGCCATGCCCGAAGGTTATGTA
CAGGAAAGAACTATATTTTTCAAAGATGACGGGAACTACAAGACACGTGCTGAAGTCAAGTTTGAAGGTGATACC
CTTGTTAATAGAATCGAGTTAAAAGGTATTGATTTTAAAGAAGATGGAAACATTCTTGGACACAAATTGGAATACA
ACTATAACTCACACAATGTATACATCATGGCAGACAAACAAAAGAATGGAATCAAAGTTAACTTCAAAATTAGACA
CAACATTGAAGATGGAAGCGTTCAACTAGCAGACCATTATCAACAAAATACTCCAATTGGCGATGGCCCTGTCCT
TTTACCAGACAACCATTACCTGTCCACACAATCTGCCCTTTCGAAAGATCCCAACGAAAAGAGAGACCACATGGT
CCTTCTTGAGTTTGTAACAGCTGCTGGGATTACACATGGCATGGATGAACTATACAAATAGGGCGCGCC][4]ACTT
CTAAATAA[GCGAATTTCTTATGATTTATGATTTTTATTATTAAATAAGTTATAAAAAAAATAAGTGTATACAAATTTT
AAAGTGACTCTTAGGTTTTAAAACGAAAATTCTTATTCTTGAGTAACTCTTTCCTGTAGGTCAGGTTGCTTTCTCAG
GTATAGTATGAGGTCGCTCTTATTGACCACACC][5]GAATTCGGATCCGTCGACCTGCAGCGT}[6]

[ ][1]: KlURA3 ORF; [ ][2]: pKlURA3 promoter; [ ][3]: pGAL1 promoter; [ ][4]: GFP(S65) ORF; [ ][5]: yADH1 terminator; { }[6]: pGAL1-GFP (with yADH terminator) from [2]; the underlined region denotes the match between the plasmid map and sequenced results.
