# Supplementary material for: Suppression of Expression Between Adjacent Genes Within Heterologous Modules in Yeast
Source: G3 (Bethesda). 2013 Nov 26;4(1):109–16. doi: 10.1534/g3.113.007922 (PMC3887525; doi:10.1534/g3.113.007922)
Supplement: Supporting Information [file supp_g3.113.007922_TableS7.pdf]

Table S7 Primers used for nucleosome scanning assay

Primer set	Control strain	divergent strain
1	FW1: CACACAAATTGGTCTTCTTTTCATCC	Same as FW1
	RV1:CAAGTCCGGTTGCATCGAAAC	Same as RV1
2	FW2: CGTAAAAGTTTCGATGCAACC	Same as FW2
	RV2: CAGTAGAGCTGAGACTCATGCAA	Same as RV2
3	FW3: GCTCTACTGGTATATGATTTTGTGG	Same as FW3
	RV3: CGTCAATACACTCCCGTCAA	Same as RV3
4	FW4: TTTGTGGACATGGTGCAACT	Same as FW4
	RV4: GCCATTTTGTGAAAGCCAGT	Same as RV4
5	FW5: GTATTGACGCTGGCGTACTG	Same as FW5
	RV5: TCTAAGATGTGGTTGTGATTGG	Same as RV5
6	FW6: GGCCCAATCACAACCACATC	Same as FW6
	RV6:GCTCATCTCAATTGATGTTATCTAAAGTC	Same as RV6
7	FW7: CAATTGAGATGAGCTTAATCATGTCAAAGC	Same as FW7
	RV7: GCTTAAGAATTGTCGTTCATGG	Same as RV7
8	FW8: GAACGACAATTCTTAAGCAAATCACGTG	Same as FW8
	RV8: CGACCTTCCATTGGATCTATATCACGT	RV8': GGGGCTCTTTACAGATCTGGATCC
9	FW9: CGTGATATAGATCCAATGGAAGG	FW9': CTTAAGCAAATCACGTGATATAGATCC
	RV9: GAGTTCTGTATTGTTCTTCTTAGTGC	RV9': GGTTTTTTTAGGCTAAGATAATGGGGC
10	FW10: CGGGATGAGCATATACAAGC	FW10': AAAGAGCCCCATTATCTTAGCC
	RV10: GCACAATAATACCGTGTAGAG	RV10': ATTACTGAAAGTTCCAAAGAGAAGG
11	FW11: GAACAATACAGAACTCTACACGG	FW11': CCTTCTCTTTGGAACTTTCAGT
	RV11: AAAACACTCGGTTTTACTCGAGC	RV11': AATATAGCAATGAGCAGTTAAGCG
Reference*	FWR: CCTTTAGCTAATAGAGTAAGCCACA	
	RVR: TTTAACACTACTGGTTTATGAAAGAAA	

*The reference primers target a constitutively nucleosomal region that is regulated independently of galactose and uracil, and thus can serve as a proper normalization standard [1].

Reference:

1. Kim HD, O'Shea EK (2008) A quantitative model of transcription factor-activated gene expression. Nat Struct Mol Biol 15: 1192-1198.
2. Longtine MS, McKenzie A, 3rd, Demarini DJ, Shah NG, Wach A, et al. (1998) Additional modules for versatile and economical PCR-based gene deletion and modification in Saccharomyces cerevisiae. Yeast 14: 953-961.
